# Monocytes and Macrophages Serve as Potent Prostaglandin D_2_ Sources during Acute, Non-Allergic Pulmonary Inflammation

**DOI:** 10.3390/ijms222111697

**Published:** 2021-10-28

**Authors:** Sonja Rittchen, Katharina Jandl, Ilse Lanz, Bernhard Reiter, Nerea Ferreirós, Daniel Kratz, Jörg Lindenmann, Luka Brcic, Thomas Bärnthaler, Reham Atallah, Horst Olschewski, Eva M. Sturm, Akos Heinemann

**Affiliations:** 1 Otto Loewi Research Center for Vascular Biology, Immunology and Inflammation, Division of Pharmacology, Medical University of Graz, 8010 Graz, Austria; sonja.rittchen@medunigraz.at (S.R.); katharina.jandl@lvr.lbg.ac.at (K.J.); ilse.lanz@medunigraz.at (I.L.); bernhard.reiter@medunigraz.at (B.R.); thomas.baernthaler@medunigraz.at (T.B.); reham.atallah@medunigraz.at (R.A.); eva.sturm@medunigraz.at (E.M.S.); 2 Ludwig Boltzmann Institute for Lung Vascular Research, 8010 Graz, Austria; horst.olschewski@medunigraz.at; 3 Pharmazentrum Frankfurt/ZAFES, Institute of Clinical Pharmacology, Goethe University Frankfurt, 60596 Frankfurt am Main, Germany; nerea.ferreiros@gmail.com (N.F.); daniel.kratz@itmp.fraunhofer.de (D.K.); 4 Department of Surgery, Divison of Thoracic and Hyperbaric Surgery, Medical University of Graz, 8010 Graz, Austria; jo.lindenmann@medunigraz.at; 5 Diagnostic and Research Institute of Pathology, Medical University of Graz, 8010 Graz, Austria; luka.brcic@medunigraz.at; 6 Department of Internal Medicine, Division of Pulmonology, Medical University of Graz, 8010 Graz, Austria; 7 BioTechMed, 8010 Graz, Austria

**Keywords:** prostaglandin D_2_, hPGDS, lipopolysaccharide, acute lung injury, mononuclear phagocytes, precision-cut lung slices

## Abstract

Acute respiratory inflammation, most commonly resulting from bacterial or viral infection, is one of the leading causes of death and disability worldwide. The inflammatory lipid mediator prostaglandin D_2_ (PGD_2_) and its rate-limiting enzyme, hematopoietic PGD synthase (hPGDS), are well-known drivers of allergic pulmonary inflammation. Here, we sought to investigate the source and role of hPGDS-derived PGD_2_ in acute pulmonary inflammation. Murine bronchoalveolar monocytes/macrophages from LPS- but not OVA-induced lung inflammation released significant amounts of PGD_2_. Accordingly, human monocyte-derived macrophages expressed high basal levels of hPGDS and released significant levels of PGD_2_ after LPS/IFN-γ, but not IL-4 stimulation. Human peripheral blood monocytes secreted significantly more PGD_2_ than monocyte-derived macrophages. Using human precision-cut lung slices (PCLS), we observed that LPS/IFN-γ but not IL-4/IL-13 drive PGD_2_ production in the lung. HPGDS inhibition prevented LPS-induced PGD_2_ release by human monocyte-derived macrophages and PCLS. As a result of hPGDS inhibition, less TNF-α, IL-6 and IL-10 could be determined in PCLS-conditioned medium. Collectively, this dataset reflects the time-dependent release of PGD_2_ by human phagocytes, highlights the importance of monocytes and macrophages as PGD_2_ sources and suggests that hPGDS inhibition might be a potential therapeutic option for acute, non-allergic lung inflammation.

## 1. Introduction

Prostaglandins are potent lipid mediators produced by various cell types upon inflammatory stimulation [1,2]. These mediators are downstream of the arachidonic acid / cyclooxygenase (COX) pathway, while rate-limiting enzymes facilitate their generation. Alongside lipocalin-type PGD synthase (LPGDS) in the central nervous system, the hematopoietic PGD synthase (hPGDS) is the rate-limiting enzyme of prostaglandin D_2_ (PGD_2_) production in peripheral tissue and is widely expressed in various immune cells [3]. Essentially, PGD_2_ has been linked to various pro-inflammatory actions, including recruitment and activation of Th2 cells [4], innate lymphoid cells [5] and granulocytes [6,7] as well as bronchoconstriction and airway hyper-reactivity in a mouse model of allergic asthma [8]. Mast cells express high levels of hPGDS and are described as the main PGD_2_ source during allergic inflammation [9,10,11].

Although less explored than allergic inflammation, a role of hPGDS and PGD_2_ expression in driving severity and pathology of acute pulmonary inflammation has been proposed [12,13]. PGD_2_ levels are significantly elevated in the bronchoalveolar fluid of mice that received intranasal bacterial lipopolysaccharide (LPS) [12]. In addition, urinary PGD_2_ metabolites have been proposed as biomarkers for acute respiratory distress syndrome (ARDS) [13] and lung sections from ARDS patients displayed an extensive immune infiltrate consisting mainly of macrophages and monocytes that stained positive for hPGDS [12]. The innate immune system, with alveolar macrophages at its center, serves as the pulmonary first-line of defense against harmful agents by recognizing damage- or pathogen-associated molecular patterns, i.e., bacterial LPS with Toll-like receptor (TLR)-4 [14,15]. Additionally, monocytes may be recruited from the circulation to either actively participate or differentiate into monocyte-derived macrophages or dendritic cells [16]. Human LPS-activated monocytes/macrophages release a plethora of inflammatory cytokines [17,18] as well as lipid mediators, i.e., prostaglandins [19,20]. In line with this, LPS-TLR-4 activation of murine bone marrow-derived macrophages upregulated COX-2 and hPGDS expression, which was accompanied by PGD_2_ release [21,22,23]. Henkel et al. showed that human monocyte-derived macrophages stimulated with IL-4 along with house dust mite allergen produce PGD_2_ [24]. In contrast, downregulation of COX-2 and prostaglandin pathway-related genes was observed in antigen-presenting cells after stimulation with IL-4/IL-13, which are typically elevated during allergic inflammation [25,26]. Of note, it has been reported that house dust mite allergen activates TLR-2 and -4 receptors on macrophages [27], which might have initiated PGD_2_ and PGE_2_ release by macrophages rather than pro-allergic stimulation.

With this background, we set out to define the role of monocytes and macrophages as PGD_2_ sources in vitro as well as in murine models of LPS-induced acute and OVA-induced allergic lung inflammation and organotypic human precision-cut lung slices. Our results suggest that mononuclear phagocytes significantly contribute to elevated PGD_2_ levels in acute, but not in established, allergic pulmonary inflammation. This information might be valuable to develop targeted anti-inflammatory therapies for acute pulmonary inflammation and respiratory distress syndrome.

## 2. Results

### 2.1. Evaluation of Hematopoietic PGD Synthase Expression in Human Peripheral Blood Leukocytes Highlights Monocytes as a Potential PGD_2_ Source

**Figure 1:**
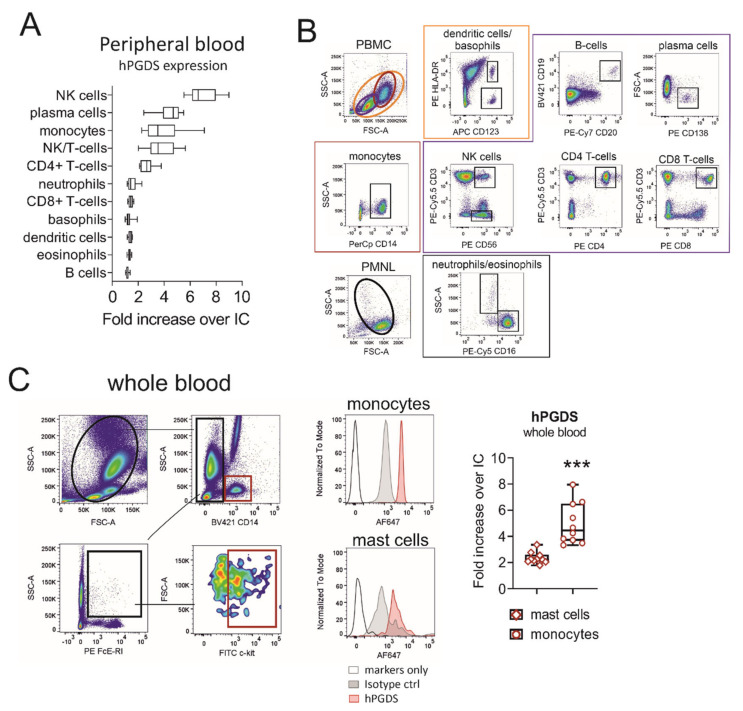
Evaluation of hematopoietic PGD synthase expression in human peripheral blood leukocytes highlights monocytes as a potential PGD_2_ source. (**A**) CD14^+^ monocytes, CD4^+^ T-cells, NK/T and NK cells as well as plasma cells stained highly positive for hPGDS. (**B**) Gating strategy for peripheral blood leukocytes, which were stained in the PMNL or PBMC fraction of peripheral blood from human donors. (**C**) For comparison, hPGDS expression in circulating mast cells and monocytes was determined in whole blood. Data are shown as box-and-whisker, *n* = 10, Student’s t-test (*** *p* < 0.001).

Our first aim was to evaluate hPGDS expression in human circulating leukocyte subsets using a flow cytometry approach. Peripheral blood leukocytes from human donors were stained in the PBMC or PMNL fraction. NK, NK/T-cells and plasma cells stained highly positive for hPGDS. In addition, about 70% of CD4^+^ T-cells and the majority of CD14^+^ monocytes expressed substantial levels of hPGDS (Figure 1A,B). No significant differences in hPGDS expression between peripheral blood immune cells from non-atopic and symptomatic atopic donors could be detected with this screening; however, monocytes, NK/T cells and CD4^+^ T-cells from atopic donors showed a slight increase in hPGDS expression (Figure S1). As mononuclear phagocytes accumulate and play a central role in modulating inflammatory reactions, we next aimed to compare hPGDS expression levels in monocytes versus Fcε-RI^+^ c-kit+ mast cells in peripheral blood, the latter being considered as prototype source of PGD_2_. Importantly, CD14^+^ monocytes displayed significantly higher hPGDS expression than mast cells from circulation (Figure 1C).

### 2.2. Monocytes and Macrophages Sorted from the Healthy Murine Lung Express hPGDS, but Not LPGDS, and Release PGD_2_ upon Stimulation with LPS/IFN-γ

**Figure 2:**
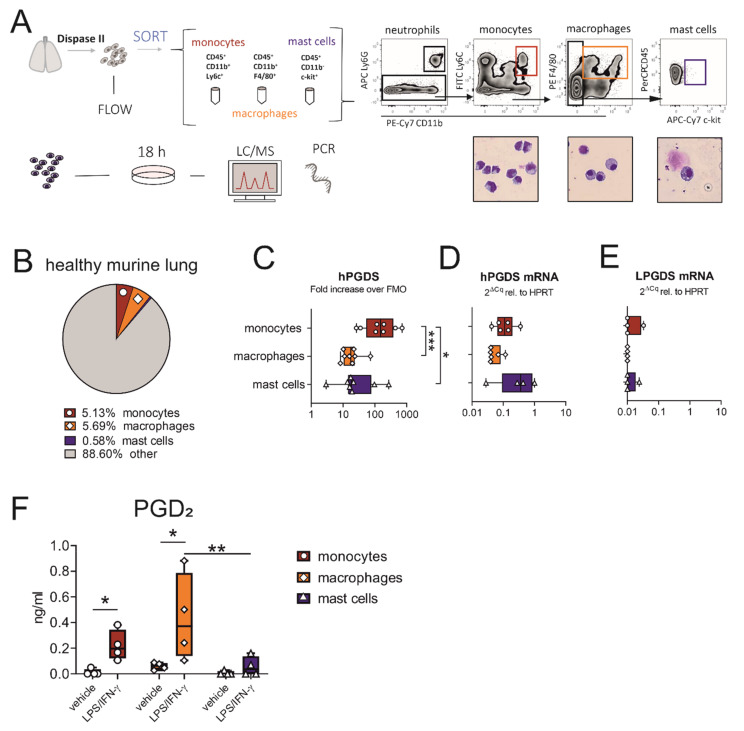
Macrophages and monocytes sorted from the healthy murine lung express hPGDS, but not LPGDS, and release PGD_2_ upon stimulation with LPS/IFN-γ. (**A**) Schematic experimental approach. Total lung was extracted and digested with Dispase II. Monocytes, macrophages and mast cells were sorted from whole lung single-cell suspension according to displayed gating strategy. Representative images of populations obtained after sorting were stained with Diff/Quik (pooled from 2 mice, *n* = 4). (**B**) Mean percentage of monocytes, macrophages and mast cells of total viable cells obtained from healthy lungs. (**C**) Flow cytometry showed that lung-associated monocytes express high levels of hPGDS. (**D**) hPGDS transcriptional expression was comparable in all three sorted populations, while (**E**) LPGDS mRNA was very low. (**F**) Stimulation with LPS/IFN-γ for 18 h significantly increased PGD_2_ levels in monocyte- and macrophage-conditioned media. Data are shown as box-and-whisker plots with one data point representing one mouse, *n* = 4–8, (**C**–**E**) one-way ANOVA for repeated measurements with Tukey’s post hoc test (log-transformed) or (**F**) two-way ANOVA for repeated measurements with Fisher’s LSD post hoc test, * *p* < 0.05, ** *p* < 0.01, *** *p* < 0.0001.

As we observed that hPGDS expression in monocytes was more prominent than in mast cells in the circulation, we next evaluated whether this was equally true for tissue-resident cells sorted from the healthy murine lung. The experimental approach is described in Figure 2A. From the whole lung single-cell suspension, we obtained the highest numbers in macrophages (~200,000–300,000), closely followed by CD14^+^ monocytes (~100,000–250,000) and only very few mast cells (~2000–10,000). The mean percentages of viable cells of each population are shown in Figure 2B. Similar to data obtained from human circulatory immune cells, hPGDS expression in pulmonary-associated monocytes exceeded expression in mast cells as demonstrated with flow cytometry (Figure 2C). Of note, hPGDS gene expression levels were comparable in all three cell types, and LPGDS levels were mostly below the detection limit (Figure 2D,E), demonstrating that hPGDS is the exclusive PGD-producing enzyme expressed in these leukocyte subsets.

When acute pulmonary inflammation was induced in C57BL/6 mice by intranasal application of 1 mg/kg LPS, a significant neutrophilic inflammation was observed, whereby also monocyte numbers were increased (Figure S2C,D, respectively). Only a few mast cells could be detected, with numbers even decreasing after LPS stimulation (Figure S2E), supporting our hypothesis that this cell type is less important as a PGD_2_ source in acute inflammation. Expression levels of hPGDS in all immune populations determined by flow cytometry and PCR were comparable in PBS- and LPS-treated mice (Figure S2G). 

To compare the roles of pulmonary-resident monocytes/macrophages and mast cells as a source for PGD_2_ in acute, non-allergic inflammation, cells sorted from healthy lungs were divided up after sorting and stimulated with bacterial LPS and interferon (IFN)-γ overnight. Indeed, prominent amounts of PGD_2_ could be determined by liquid chromatography/mass spectrometry (LC/MS) in monocyte and especially macrophage-conditioned medium, but only low amounts in mast cell-conditioned medium after stimulation (Figure 2F). Both populations, monocytes and macrophages, yielded between 5–10 ng PGD_2_/mL after stimulation for 18 h, calculated for a million cells and a volume of 1 mL (Figure S3A). Besides PGD_2_, PGE_2_ was strongly upregulated in monocyte- and macrophage-conditioned medium, TBX_2_ levels were elevated, while PGF_2α_ was unchanged (Figure S3B–D). This suggests that during LPS-induced inflammation, pulmonary associated monocytes and resident macrophages should also be able to contribute high levels of PGD_2_ and potentially other lipid mediators, while mast cells are present in low numbers and show a minor response to acute stimuli such as LPS.

### 2.3. Broncho-Alveolar Mononuclear Phagocytes from LPS- but Not OVA-Challenged Mouse Lungs Release High Amounts of PGD_2_

**Figure 3:**
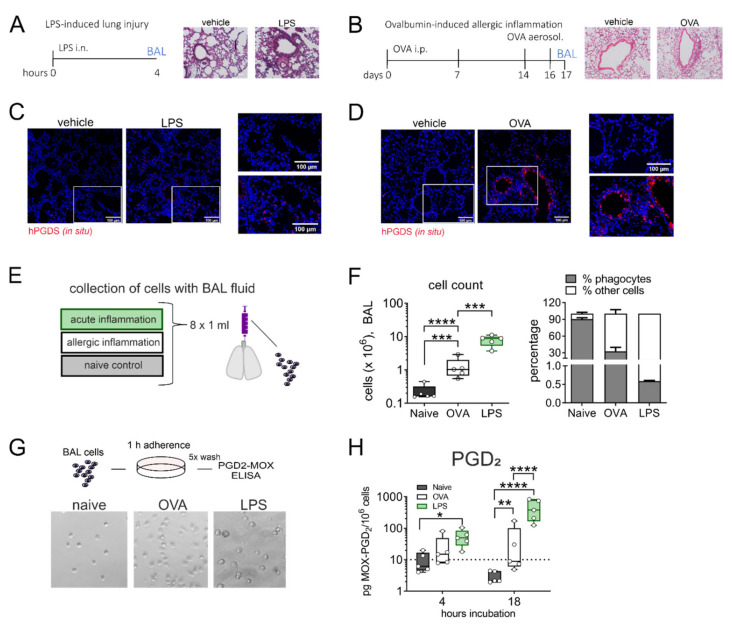
Alveolar monocytes/macrophages from LPS- but not OVA-challenged mouse lungs release high amounts of PGD_2_. (**A**) Experimental protocol of LPS-induced lung inflammation and (**B**) OVA-induced allergic inflammation in BALBc mice. Lung histology for both pulmonary inflammation models are shown (H&E staining, representative of 3 mice each). (**C**,**D**) In both inflammatory models, hPGDS mRNA was notably upregulated (representative of 3 mice, scale bar 100 µm). (**E**) Alveolar cells were collected by broncho-alveolar lavage (BAL) at indicated time points. (**F**) BAL fluid from LPS-induced lung inflammation and OVA-induced allergic inflammation showed a characteristic (granulocytic) infiltration. The percentage of mononuclear phagocytes in BALF was estimated by morphological differences. (**G**) BAL cells were seeded into CellBind plates and non-adherent cells washed off after 1 h adherence. Enriched adherent phagocytes were incubated for indicated time points. Representative images of obtained populations are shown. (**H**) Mononuclear phagocytes from LPS-induced lung inflammation released significantly more PGD_2_ after 18 h in culture. Data are shown as bar chart + SEM or box-and-whisker plot, *n* = 5. One-way ANOVA with Tukey‘s post hoc test (log-transformed) (**F**) or two-way ANOVA with Tukey’s post hoc test (log-transformed) (H), * *p* < 0.05, ** *p* < 0.01, *** *p* < 0.001, **** *p* < 0.0001.

Given the strong release of PGD_2_ by lung resident monocytes and macrophages in response to LPS/IFN-γ stimulation in vitro, we next aimed to transfer our findings to an in vivo setting. It has been reported frequently that PGD_2_ is elevated in bronchoalveolar lavage fluid (BALF) in asthmatics and is a known driver of allergic inflammation [28]. Still, its role in acute lung injury is less established even though PGD_2_ was also more abundant in BALF of mice that received intranasal LPS [12]. Thus, we directly compared the extent of PGD_2_ production by alveolar mononuclear phagocytes in two different murine models: LPS-induced acute lung inflammation and ovalbumin (OVA)-induced allergic lung inflammation (experimental protocol in Figure 3A,B). Essentially, overall leukocyte infiltration and plasma extravasation around airways and vessels in both models was comparable. In situ hybridization revealed an increase in hPGDS mRNA expressing cells in both models. In LPS lungs, hPGDS transcription was induced primarily in the parenchyma and infiltrating cells and occasionally in epithelial cells (Figure 3C). In contrast, OVA challenge resulted in a prominent increase of hPGDS expression in bronchial epithelial cells primarily (Figure 3D). Broncho-alveolar cells were collected with BAL fluid from both models (experimental setup Figure 3E). The highest increase in BALF cell number could be observed in LPS-induced lung inflammation. The percentage of mononuclear cells in BALF was determined using a morphological approach, i.e., cell size and nucleus morphology. In naïve mice, the mononuclear phagocyte fraction comprised about 90% of total cells, while in BALF obtained from OVA and LPS models the percentage was significantly lower, indicating a tremendous lymphocyte and granulocyte influx (Figure 3F). Broncho-alveolar mononuclear phagocytes enriched from BAL fluid (representative images shown in Figure 3G) from mice that received intranasal LPS produced significant amounts of PGD_2_ after 18 h incubation without further stimulation; in contrast, cells from OVA-induced allergic inflammation released almost no PGD_2_ (Figure 3H). This confirms that in non-allergic inflammation, e.g., initiated by bacterial LPS, mononuclear phagocytes contribute significant amounts of PGD_2_.

### 2.4. Human Monocytes and Monocyte-Derived Macrophages Express Robust Basal Levels of hPGDS, whereby LPS/IFN-γ Upregulates COX-2 and hPGDS

**Figure 4:**
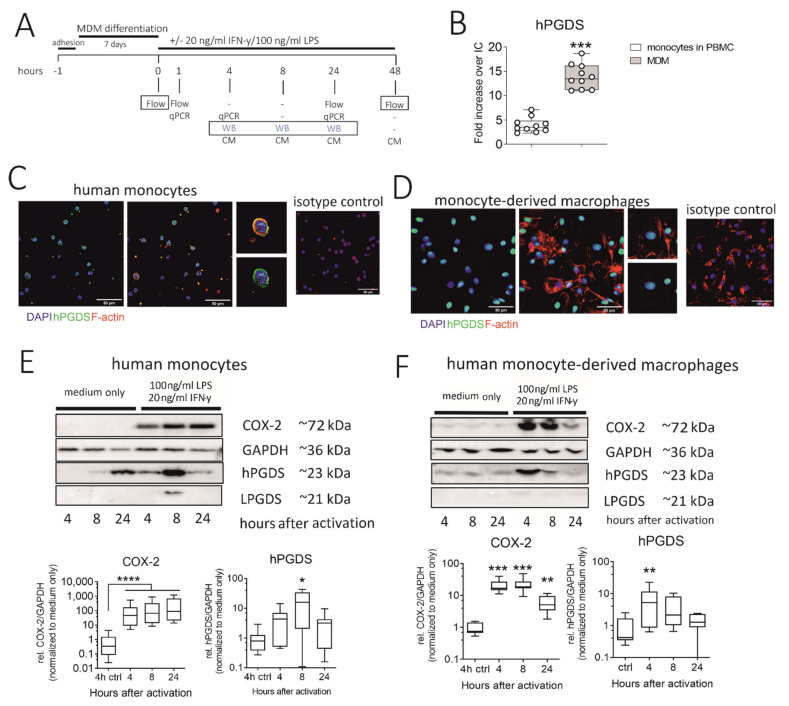
Human monocytes and monocyte-derived macrophages constitutively express hPGDS, which is upregulated in parallel to COX-2 by LPS/IFN-γ. (**A**) Peripheral blood monocytes were either directly activated or first differentiated into monocyte-derived macrophages (MDM). hPGDS expression was evaluated by flow cytometry, microscopy or Western blotting at indicated time points. (**B**) As evaluated by flow cytometry, MDM express higher levels of hPGDS compared to monocytes in PBMC (*n* = 10). (**C**) Peripheral blood monocytes and (**D**) MDM express high basal hPGDS levels as determined by fluorescence microscopy (*n* = 3 donors, scale bar 50 µm). COX-2 and hPGDS densitometric results obtained from (**E**) monocyte and (F) macrophage lysates by Western blotting were normalized to GAPDH values and subsequently to unstimulated controls (*n* = 7–8). Two-way ANOVA for repeated measurements with Dunnetts’s post hoc test (log-transformed), * *p* < 0.05, ** *p* < 0.01, *** *p* < 0.001, **** *p* < 0.0001.

We next explored the regulation and timing of PGD_2_ release in human monocytes and macrophages. Our experimental approach is summarized in Figure 4A. As determined by flow cytometry, in vitro differentiated monocyte-derived macrophages (MDM) expressed significantly more hPGDS than peripheral blood monocytes (Figure 4B). However, immunofluorescence staining revealed that already at basal state, both human monocytes and monocyte-derived macrophages express substantial levels of hPGDS (Figure 4C,D, respectively). Notably, hPGDS expression was unchanged in monocytes and macrophages after 24 h stimulation with LPS/IFN-γ, as determined by flow cytometry (Figure S4B,D). At 48 h after activation with LPS/IFN-γ or IL-4, human MDM were stained with anti-CD80 for pro-inflammatory and anti-CD206 antibodies for an anti-inflammatory polarization to show activation by LPS/IFN-γ and IL-4, respectively (Figure S4E). Interestingly, we observed reduced hPGDS expression in pro-inflammatory macrophages (Figure S6E). This was possibly already the late phase after LPS/IFN-γ activation, and we examined hPGDS expression at two earlier time points by Western blotting. Indeed, this confirmed a rapid COX-2 upregulation in monocytes and macrophages as early as 4 h after LPS/IFN-γ activation. In monocytes, hPGDS was significantly upregulated on protein level 8 h after activation and in macrophages after 4 h (Figure 4E,F). LPGDS could not be detected in most monocyte and macrophage protein lysates. This further strengthens the hypothesis that COX-2/hPGDS is the major mechanism of PGD_2_ production in primary human mononuclear phagocytes.

### 2.5. LPS/IFN-γ, but Not IL-4, Initiates PGD_2_ Production by Human Monocytes/Macrophages, Which Precedes a More Extensive PGE_2_ Release 

**Figure 5:**
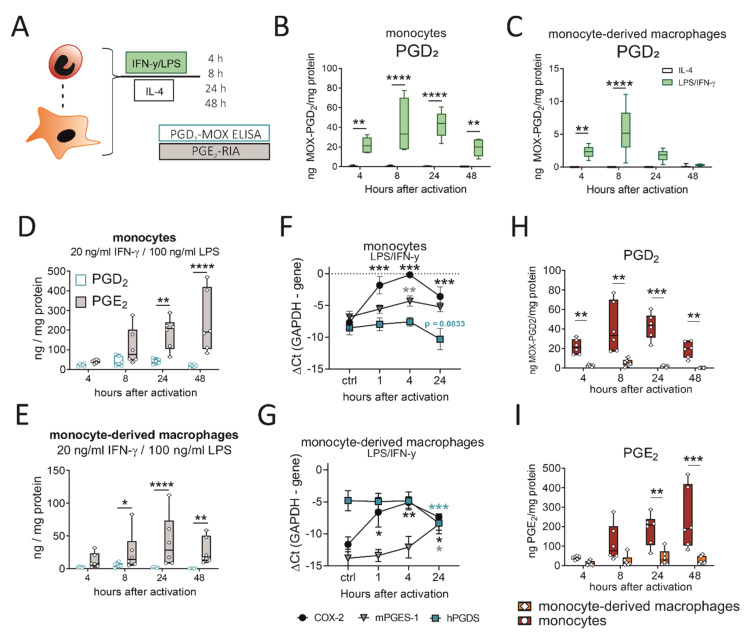
LPS/IFN-γ initiates PGD_2_ release by human monocytes/macrophages, while PGD_2_ peaks before PGE_2_ and monocytes surpass macrophages as a PG source. (**A**) Experimental setup. (**B**) Activation of human peripheral blood monocytes or (**C**) monocyte-derived macrophages with LPS/IFN-γ, but not IL-4, induced significant PGD_2_ release. PGD_2_ precedes a more extensive release of PGE_2_ by (**D**) monocytes and (**E**) macrophages after activation. Transcriptional regulation of COX-2, hPGDS and mPGES-1 in (**F**) monocytes and (**G**) macrophages after LPS/IFN-γ. Human monocytes surpass monocyte-derived macrophages as (**H**) PGD_2_ as well as (**I**) PGE_2_ sources after LPS/IFN-γ activation. Data are shown as box-and-whisker plot (*n* = 6) or line (*n* = 6–7; mean + SEM). Two-way ANOVA for repeated measurements with Sidak‘s post hoc test (**B**–**D**,**E**,**H**,**I**) or one-way ANOVA for repeated measurements with Dunnett’s post hoc test (**F**,**G**, difference to control), * *p* < 0.05, ** *p* < 0.01, *** *p* < 0.001, **** p < 0.0001.

So far, we could show that murine monocytes and macrophages express hPGDS and release significant amounts of PGD_2_ in acute LPS-induced, but not OVA-induced, allergic inflammation. Next, we aimed to confirm that these observations also apply to human peripheral blood monocytes and monocyte-derived macrophages. Cells were either activated with LPS/IFN-γ, as classical type-1 inflammatory stimulus, or IL-4, to simulate a type-2 cytokine-driven inflammation, which is characteristic in allergic reactions. Indeed, we detected significant amounts in the ng/mg protein-range of PGD_2_ in conditioned medium from both phagocyte populations after LPS/IFN-γ, but not IL-4 stimulation (Figure 5B,C). These observations are in line with what we observed for murine pulmonary leukocytes (Figure 3) and show that also in human monocytes and macrophages PGD_2_ production is triggered by LPS/IFN-γ, but not IL-4. Notably, PGD_2_ release by monocytes and monocyte-derived macrophages peaked between 8 to 24 h after pro-inflammatory activation, which is highly correlated with hPGDS protein levels (Figure 4). As neutrophils are present in great numbers during acute inflammation, we additionally investigated PGD_2_ production by human peripheral blood neutrophils; however, circulatory neutrophils neither expressed relevant levels of hPGDS, nor released PGD_2_ after LPS stimulation (Figure S5). Previously, we have shown that primary human monocytes release PGE_2_ after LPS/IFN-γ stimulation [20,29]. In line with this, we observed a steady increase in PGE_2_ levels within 48 h that exceeded PGD_2_ levels at all time points (Figure 5D,E). PGE_2_ levels in monocyte- and macrophage-conditioned medium suggested that both populations release low but detectable levels of PGE_2_, while IL-4 stimulation did not change observed values (Figure S6A,B). Higher levels of PGE_2_ compared to PGD_2_ could be measured in conditioned medium from unstimulated monocytes/macrophages and when activated with IL-4 (Figure S6C,D). We conclude that LPS/IFN-γ activation of primary human monocytes and macrophages initiates both PGD_2_ and PGE_2_ release. PGD_2_ precedes PGE_2,_ but its secretion declines 24 to 48 h after stimulation, most likely due to a negative feedback loop resulting in the downregulation of hPGDS mRNA. At the same time, mPGES-1 is unchanged (or even increased in macrophages) (Figure 5F,G).

### 2.6. Human Monocytes Surpass Monocyte-Derived Macrophages as PGD_2_ and PGE_2_ Sources after LPS/IFN-γ

Our experimental setup enabled us to directly compare prostaglandin production by human monocytes and monocyte-derived macrophages after pro-inflammatory stimulation. Remarkably, human monocytes released significantly higher levels of PGD_2_ (Figure 5H) as well as PGE_2_ (Figure 5I) in comparison to monocyte-derived macrophages. In a monocyte-conditioned medium, we could measure up to 50 ng MOX-PGD_2_ / mg protein values at 8 and 24 h after activation, while PGD_2_ levels declined after 48 h. This highlights human monocytes as effector cells, which may even surpass monocyte-derived macrophages as prostaglandin sources.

### 2.7. Inhibition of hPGDS Prevents PGD_2_ Production by Human Monocyte-Derived Macrophages, while CD68^+^ Cells in Human Lung Slices Constitutively Express hPGDS

**Figure 6:**
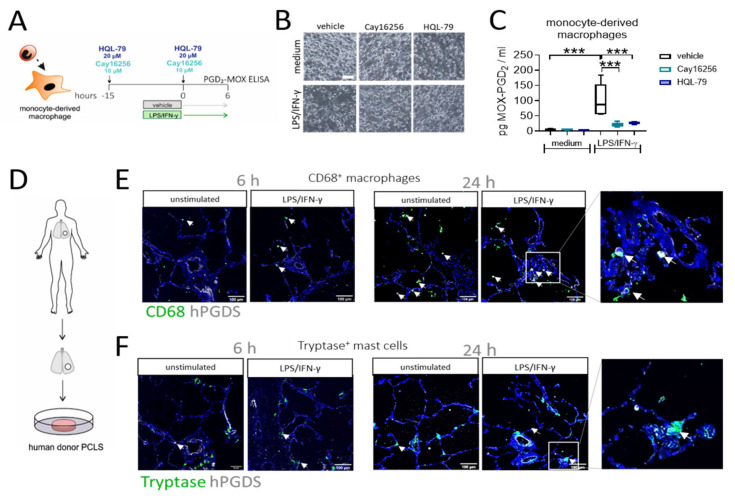
Inhibition of hPGDS prevents PGD_2_ production by human monocyte-derived macrophages and CD68^+^ phagocytes strongly express hPGDS in human lung tissue. (**A**) Human monocyte-derived macrophages were incubated with hPGDS inhibitors or vehicle overnight and stimulated with LPS/IFN-γ in the presence of inhibitors for 6 h. (**B**) Monocyte-derived macrophages at the time of collection (representative of *n* = 4 donors; scale bar 100 µm). (**C**) hPGDS inhibitors Cay16256 (10 µM) and HQL-79 (20 µM) significantly reduced PGD_2_ production by macrophages. (**D**) Serial cut precision-cut lung slices (PCLS) from human lung tissue resections were used. (**E**) Co-staining of CD68 and hPGDS showed that macrophage-like cells already express hPGDS in unstimulated PCLS and to a stronger extent in LPS/IFN-γ-treated PCLS. (**F**) hPGDS^+^ tryptase^+^ mast cells were present in donor PCLS, but hPGDS levels were less pronounced compared to macrophages and unaffected by stimulation with LPS/IFN-γ. Data are shown as box-and-whisker plot, *n* = 5. One-way ANOVA for repeated measurements (mixed model) with Sidak’s post hoc test, *** *p* < 0.001.

To confirm that macrophage-derived PGD_2_ is indeed dependent on hPGDS and to explore the potential of pharmacological blockade of macrophage-derived PGD_2_ production, we generated human monocyte-derived macrophages, pre-treated the cells with two commercially available hPGDS inhibitors, Cay16256 and HQL-79, at indicated concentrations, followed by stimulation with LPS/IFN-γ for 6 h (Figure 6A). Applied concentrations of hPGDS inhibitors did not affect the cellular integrity of MDM in culture (Figure 6B). Still, they significantly diminished MOX-PGD_2_ amounts detectable in MDM-conditioned medium after LPS/IFN-γ activation (Figure 6C).

To finally prove the role of monocytes/macrophages as a source for PGD_2_ in acute non-allergic pulmonary inflammation in human lung, we performed an ex vivo approach using human donor precision-cut lung slices stimulated with LPS/IFN-γ or IL-4/IL-13, respectively. Importantly, immunofluorescence staining confirmed the presence of both CD68^+^ macrophages and tryptase^+^ mast cells, in sections of unstimulated human lung tissue (Figure 6E,F). In line with previous observations [30], macrophages stained highly positive for hPGDS, while expression levels in mast cells were lower. Furthermore, a stronger hPGDS signal was visible in CD68^+^ cells in PCLS stimulated with LPS/IFN-γ, but not exclusively, as also epithelial cells showed enhanced hPGDS expression after 24 h. In contrast, mainly bronchial epithelial cells upregulated hPGDS in sections that were incubated with IL-4/IL-13, although macrophages stained positive, too (Figure S7). Conclusively, human MDM produce PGD_2_ in an hPGDS-dependent manner, which could be successfully blunted via hPGDS inhibition. In human lung tissue sections, resident macrophage populations strongly expressed hPGDS and, thus, have the machinery to supply PGD_2_ during inflammation. 

### 2.8. LPS/IFN-γ, but Not IL-4/IL-13, Induces hPGDS-Dependent PGD_2_ Release and Cytokine Secretion by Human Donor Precision-Cut Lung Slices

**Figure 7:**
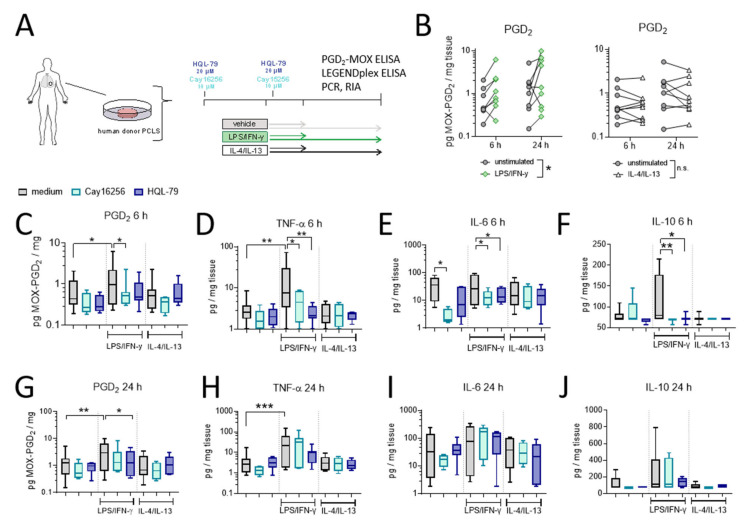
LPS/IFN-γ, but not IL-4/IL-13, induce hPGDS-dependent PGD_2_ release and cytokine secretion by human donor precision-cut lung slices. (**A**) Serial precision-cut lung slices (PCLS) from human donors were pre-treated and stimulated in presence of hPGDS inhibitors HQL-79 (20 µM) or Cay16256 (10 µM) for indicated durations and activated with LPS/IFN-γ (100 ng/mL and 20 ng/mL, respectively) or IL-4/IL-13 (20 ng/mL). (**B**) LPS/IFN-γ, but not IL-4/IL-13, initiated a significant release of PGD_2_. (**C**) Inhibition of hPGDS significantly reduced PGD_2_ release as well as (**D**) TNF-α, (**E**) IL-6 and (**F**) IL-10 secretion by PCLS after LPS/IFN-γ stimulation for 6 h. After 24 h stimulation with LPS/IFN-γ, HQL-79 still significantly reduced (**G**) PGD_2_ and (**H**) TNF-α release. There were no significant changes in (**I**) IL-6 and (**J**) IL-10 levels. Data are shown as box-and-whisker or before-after plots, *n* = 5–9. (**B**) Two-way ANOVA for repeated measurements (mixed model), (**C**–**E**,**G**,**H**) One-way ANOVA for repeated measurements (mixed model) with Sidak’s post hoc test (log-transformed), (**F**,**J**) Kruskal–Wallis test, * *p* < 0.05, ** *p* < 0.01, *** *p* < 0.001.

In the final set of experiments, we evaluated the extent of hPGDS-dependent PGD_2_ release using human organotypic lung slices following stimulation with LPS/IFN-γ and IL-4/IL-13, respectively (experimental approach is depicted in Figure 7A). We observed a significant PGD_2_ release already in the first 6 h after activation in LPS/IFN-γ, but not IL-4/IL-13 stimulated PCLS (Figure 7B). This falls in line with what we observed for human mononuclear phagocytes, where PGD_2_ levels in conditioned medium reached a maximum approximately 8 h after activation (Figure 5). Notably, PGE_2_ levels were also elevated after LPS/IFN-γ stimulation of human PCLS and were present at significantly higher amounts compared to PGD_2_ at both time points (Figure S8). PCR and Western blotting of PCLS tissue lysates confirmed a marked upregulation of COX-2 after LPS/IFN-γ at both time points. At the same time, hPGDS mRNA and protein expression and mPGES-1 mRNA levels were not significantly altered in total tissue (Figure S9). Importantly, PGD_2_ release was blunted by the presence of hPGDS inhibitors Cay16256 or HQL-79 (Figure 7C,G); moreover, PGE_2_ levels were slightly reduced by both hPGDS inhibitors, but significantly so by HQL-79 at the 6 h time point (Figure S8B). Next, we evaluated whether inhibition of PGD_2_ production could affect the production of pro-inflammatory cytokines in human lung tissue. Remarkably, both hPGDS inhibitors ablated TNF-α, IL-6 and IL-10 release by PCLS in the first 6 h after LPS/IFN-γ activation (Figure 7 D–F), which was not the case for conditioned medium collected 24 h after activation (Figure 7H–J). These observations could be partially attributed to respective changes in transcriptional activity: TNF-α and IL-6 mRNA levels were less potently induced in PCLS that received hPGDS inhibitors after 6 h, but not 24 h (Figure S10A,B, respectively). Notably, COX-2 mRNA expression resembled the pattern observed for those two cytokines (Figure S10). Additional inflammatory cytokines were measured and showed similar tendencies without significant changes (Figure S11). These data further strengthen the notion that hPGDS-derived PGD_2_ contributes to acute pulmonary inflammation in a human setting and offers a potential new approach to specifically attenuate a cytokine storm. 

## 3. Discussion

While mast cells have been considered the primary PGD_2_ source in allergic inflammation, it is still unclear which immune cells express the hematopoietic PGD synthase, the rate-limiting enzyme of PGD_2_ production, and produce PGD_2_ during acute inflammation, i.e., bacterial or viral infection. In this study, we showed that human and murine monocytes and macrophages express hPGDS and contribute significant levels of PGD_2_ in the early phase of acute, but not allergic, pulmonary inflammation. Most importantly, inhibition of hPGDS-catalyzed PGD_2_ production upon LPS activation in human precision-cut lung slices restricted pro-inflammatory cytokine secretion and, thus, revealed a novel therapeutic target for uncontrolled acute lung inflammation. 

In lung sections of ARDS patients, we observed that a majority of hPGDS^+^ cells had mononuclear phagocyte morphology [12]. During inflammation, monocyte-derived macrophages sometimes outnumber tissue-resident macrophages and modulate the inflammatory response and outcome [31]. Indeed, CD14^+^ monocytes lined up third in our flow cytometry screen to evaluate hPGDS expression. Considering that resident cell populations in the lung often differ in phenotype and function from circulating populations [32], we showed that monocytes/macrophages expressed even higher levels of hPGDS than mast cells in the healthy murine lung and are present at significantly higher numbers. Interestingly, the second PGD_2_-related enzyme, LPGDS, was present at much lower levels or even absent in murine and human monocytes/macrophages. This is in line with another study that detected strong hPGDS, but hardly any LPGDS, expression in murine peritoneal macrophages [23]. Furthermore, hPGDS but not LPGDS-targeting siRNA, as well as co-treatment with HQL-79, an hPGDS inhibitor, but not AT-56, an LPGDS inhibitor, attenuated PGD_2_ production in mouse bone marrow-derived macrophages [22]. Microbial products, including bacterial LPS, have been shown to induce PGD_2_ production in murine bone marrow-derived and peritoneal macrophages by TLR activation [21,23]. Notably, human peripheral blood monocytes react strongly to LPS activation and release high levels of PGE_2_ [20], which shares most of its generation pyramid with PGD_2_. In human monocytes/macrophages, hPGDS was significantly upregulated in parallel with COX-2 upon LPS/IFN-γ activation. Collectively, this indicates that PGD_2_ generation by human monocytes/macrophages is likely COX-2/hPGDS-dependent.

In both acute and allergic lung inflammation, monocytes/macrophages have been considered central players of pathological inflammatory states [12,33]. In our preceding study, we saw that PGD_2_ was elevated in the bronchoalveolar fluid of mice that received intranasal LPS [12], which was a novel observation, compared to the very well explored elevated PGD_2_ levels in BAL fluid of patients suffering from allergic asthma [28] and allergic lung inflammation in mice [34,35]. Sensitization and challenge with ovalbumin generates a potent type-2-biased inflammatory response, amongst others a strong induction of IL-4 and IL-13 in lung and BAL fluid [36,37,38]. Unlike LPS, though, stimulation of human monocytes and macrophages with IL-4 or in vivo challenge with type-2 cytokine-inducing ovalbumin of murine bronchoalveolar, mononuclear phagocytes did not initiate prostaglandin production. However, we cannot exclude that macrophages release PGD_2_ earlier during OVA sensitization or challenge. It has been proposed that short exposure (24 h) of human monocyte-derived macrophages to house dust mites, a major allergen, initiates PGD_2_ release [24]. In this case, PGD_2_ production in macrophages might be initiated in a similar way to LPS, though, as it has been reported that macrophage immune response to house dust mites is mediated through TLR-2 and -4 [27].

In vitro LPS/IFN-γ stimulation initiated the release of significant amounts of PGD_2_ by sorted murine, lung-resident monocytes and macrophages, whereas in mast cell-conditioned medium hardly any PGD_2_ could be detected. Intranasal LPS raised pulmonary monocyte counts while reducing macrophage numbers in mice. This is an observation we and others have reported in previous studies [12,39], which supports the involvement of monocytes and monocyte-derived cells in the inflammatory process. Indeed, our data clearly showed a potent PGD_2_ release by bronchoalveolar mononuclear phagocytes, consisting of alveolar macrophages, monocytes and monocyte-derived macrophages, enriched from LPS-lungs. When human monocytes or monocyte-derived macrophages were cultured in the presence of LPS/IFN-γ, both cell types released significant amounts of PGD_2_. Until now, monocytes have not been considered as a relevant PGD_2_ source, but these findings suggest they are. Conclusively, this suggests that mononuclear phagocytes are activated in the early stages of acute inflammation, i.e., initiated by bacterial LPS, and contribute significant levels of PGD_2_, modulating inflammation in return, while in an established, allergic environment, other cell types, i.e., mast cells or epithelial cells, are possibly more important.

PGD_2_ and PGE_2_ often act as opposing mediators in pulmonary inflammation, whereby a lower PGD_2_-to-PGE_2_ ratio correlates with a better prognosis for respiratory inflammation [40]. Consistent with hPGDS protein expression, PGD_2_ was released early and peaked between 8 and 24 h after activation, while PGE_2_ surpassed PGD_2_ levels in monocyte/macrophage-conditioned medium at later time points. This is in line with another study showing that LPS-activated murine bone marrow-derived macrophages release PGD_2_ in the early phase (up to 8 h after activation), while PGE_2_ release increased and exceeded PGD_2_ levels after 24 h [21]. Here, we could show that this is also true for human monocytes and monocyte-derived macrophages. Essentially, this suggests a possible negative feedback loop causing downregulation of hPGDS, but not mPGES-1 mRNA expression, after an acute response, and is potentially critical for inflammatory resolution. 

In recent years, organotypic human precision-cut lung slices have been well established as a model for acute and/or allergic pulmonary inflammation [41,42,43]. Danov et al. described human PCLS stimulated with IL-13 as a robust model for allergic inflammation and confirmed potent mucus and type-2 cytokine production [43]. Furthermore, stimulation of human PCLS with LPS successfully induced the production of pro-inflammatory mediators including TNF-α, IL-6, IL-1β, IL-10 and IL-8 [41,44], which was prevented by co-incubation with dexamethasone [41]. In this study, bacterial LPS/IFN-γ, but not IL-4/IL-13, as representative allergic stimuli, induced a significant upregulation of COX-2 and PGD_2_ release in human PCLS. Notably, resident CD68^+^ macrophage populations express hPGDS at even higher levels than mast cells, as estimated by immunofluorescence staining, emphasizing that resident macrophages likely contribute to elevated PGD_2_ levels in acute lung inflammation. In PCLS tissue lysates, neither mRNA nor protein levels of hPGDS were significantly regulated after LPS/IFN-γ. A reason why hPGDS protein was induced in macrophages/monocytes, but not in PCLS lysates, could be that overall hPGDS expression in tissue does not represent hPGDS expression in individual cell populations, e.g., too few macrophages were present to see an overall increase. Interestingly, no significant change in PGD_2_ (or PGE_2_) could be observed between 6 h and 24 h, which, in combination with monocyte/macrophage data, may point toward phagocytes contributing to the early release, while at later time points other cell types may take over, e.g., epithelial cells, as has been observed in a study investigating viral exacerbations of asthma [34]. 

Both hPGDS inhibitors used in our study, HQL-79 and Cay16256, are orally bioavailable and their therapeutic potential against allergic airway inflammation was indicated in various studies [45,46,47]. HQL-79 significantly reduced PGD_2_ levels in BAL fluid collected 10 min after OVA-induced allergic airway inflammation, i.e., in the early phase of antigen challenge [45]. However, not much has been reported on hPGDS inhibition during acute inflammation yet. Here, we saw a successful reduction of PGD_2_ in the first 6 h, but not after 24 h of LPS/IFN-γ stimulation, which was reflected by pro-inflammatory cytokine levels. Importantly, targeted pharmacological blockade of hPGDS-dependent PGD_2_ production in PCLS significantly reduced the production of pro-inflammatory cytokines, including TNF-α, IL-6 and IL-10. It has been reported that DP1 receptor activation induces potent TNF-α release by monocytes, neutrophils and also epithelial cells [48], hence, there might be an autocrine stimulation of monocytes/macrophages involved in this process. In parallel with a significant reduction of PGD_2_, we could determine lower PGE_2_ levels in hPGDS-inhibitor-treated PCLS. This might result from reduced COX-2 mRNA levels and pro-inflammatory cytokines due to hPGDS/PGD_2_ inhibition and, in turn, would not jeopardize inhibitor specificity. There could be several modulating factors requiring further research. However, inhibitor half-life might be a crucial factor for the optimal route of administration and treatment scheme at a later stage. 

Regarding limitations, questions about the therapeutic potential remain and further investigation will be required to establish the benefits of hPGDS inhibition in pulmonary disease. Of specific interest will be balancing beneficial effects, e.g., reduced neutrophil influx [12], COX-2 expression and inflammatory cytokine release (as shown in this study), with potential detrimental effects, e.g., loss of endothelial barrier enhancement [49] and PGD_2_-DP1 mediated anti-viral response [34]. 

To conclude, our study suggests that averting the release of hPGDS-derived PGD_2_ is a promising approach to limit a cytokine storm and encourages further research to explore potential benefits in different varieties of acute pulmonary inflammatory disorders. 

## 4. Materials and Methods

### 4.1. Ethical Approvals 

All experiments involving primary cells from human subjects were approved by the Institutional Review Board of the Medical University of Graz (EK 17–291 ex 05/06). All volunteers signed an informed consent. The collection of human lung samples was approved by the Institutional Ethics Board (32–446 ex 19/20), following the patients’ informed consent. All studies involving animal experiments were approved by the Animal Ethics Committee of the Austrian Federal Ministry of Science and Research and carried out in line with the European Community’s Council Directive: OVA-induced allergic inflammation model with BALB/c mice (BMWF-66.010/0094- II/3b/2013), LPS-induced pulmonary inflammation model with BALB/c mice (BMWFW-66.010/0046- II/3b/2013) and LPS-induced mouse model with C57BL/6 (Amendment to protocol number: BMWFW-66.010/0119-V/3b/2018).

### 4.2. Preparation of Human Peripheral Blood Leukocytes

Human polymorphonuclear leukocytes (PMNL) and peripheral blood mononuclear cells (PBMC) were isolated from citrated blood by density gradient sedimentation as previously described [50]. For qPCR and immunofluorescence staining, monocytes were enriched using a Monocyte Isolation Kit II (Miltenyi Biotech, Bergisch Gladbach, Germany) according to the manufacturer’s instructions.

### 4.3. Generation of Human Monocyte-Derived Macrophages

Peripheral blood monocytes in the PBMC fraction were resuspended in pre-warmed adhesion medium (RPMI supplemented with 5% human AB serum, non-essential amino acids, sodium pyruvate, HEPES and 1% penicillin/streptomycin) at a concentration of 10 Mio cells/mL and seeded onto Corning^®^ CellBIND plates (Sigma-Aldrich, St. Louis, Missouri, USA) for 1.5 h at 37 °C in a humidified atmosphere with 5% CO_2_. Non-adherent cells were washed off and enriched monocytes incubated with differentiation medium (10% FCS, 1% penicillin/streptomycin and 20 ng/mL rh M-CSF (Peprotech, New Jersey, USA, #AF-300)) for 6 to 8 days. Human MDM were activated with 20 ng/mL recombinant human (rh) IFN-γ (Immunotools, Friesoythe, Germany #11343534) and 100 ng/mL LPS (Sigma-Aldrich, #L2880) or 20 ng/mL rh IL-4 (Immunotools, #11340043). To ensure successful activation, accutase-detached macrophages were stained with PE-labelled monoclonal antibodies for CD80 (20 µg/mL, PE-conjugated, BD Biosciences, Franklin Lakes, New Jersey, USA, #557227) and CD206 (20 µg/mL, PE-conjugated, BD Bioscience, #555954) or respective isotype controls (20 μg/mL, PE-conjugated) for 30 min. 

### 4.4. Precision-Cut Lung Slices

The collection of human lung samples was approved by the Institutional Ethics Board (32-446 ex 19/20), following the informed consent of the patients. Fresh lung tissue was obtained from tumor resections. Only adjacent, non-tumorous tissue was used for the experiments. Briefly, cylindrical cores (8 mm in diameter) were sliced (sections of 200 μm ± 20 μm) using a Krumdieck live tissue microtome in Earle’s balanced salt solution containing 25 mmol HEPES and 17 mmol glucose. Media was changed to incubation medium, additionally containing sodium-pyruvate, MEM amino acids, MEM vitamins and L-glutamine (all ThermoFisher Scientific, Waltham, Massachusetts, USA) and penicillin/streptomycin, and incubated in a humidified atmosphere with 5% CO_2_ at 37 °C. The incubation medium was changed at least 5 times after 45-min wash periods. hPGDS inhibitors were added at indicated concentrations (10 µM Cay16256 [10 mM stock in DMSO]; 20 µM HQL-79 [10 mM stock in MeOH], Cayman Chemicals, Ann Arbor, Michigan, USA) and incubated overnight. Media were changed in the morning and respective wells received freshly prepared media containing hPGDS inhibitors. PCLS were either left untreated or activated with 100 ng/mL LPS and 20 ng/mL IFN-γ or 20 ng/mL rh IL-4 and 20 ng/mL rh IL-13. Conditioned media and tissue were collected 6 h or 24 h after activation. The wet weight of PCLS was recorded and the tissue was either snap-frozen in liquid nitrogen for RNA and protein or fixed with formalin for 1 h at room temperature and embedded in paraffin.

### 4.5. LPS-Induced Lung Inflammation Model

Acute pulmonary inflammation was induced by intranasal application of 1 mg LPS/kg body weight in 3-month-old BALB/c mice for BAL collection. Prior to LPS application, mice were lightly anesthetized by intraperitoneal injection of 120 µL ketamine/xylazine solution (60 mg/kg body weight Ketasol (aniMedica, OGRIS Pharma V, Wels, Austria), 6 mg/kg body weight Rompun (Bayer, Vienna, Austria) in saline solution). LPS solution was added dropwise to the nostrils of mice to be inhaled. Broncho-alveolar fluid or lung was collected 4 h after LPS application. For myeloid cell sorting from whole lung single-cell suspension, lungs were collected from 3-month-old C57BL/6j mice with or without prior intranasal LPS or PBS instillation.

### 4.6. Ovalbumin-Induced Allergic Airway Inflammation

Allergic lung inflammation was induced in 3-month-old BALB/c mice (*n* = 5) by immunization with ovalbumin (OVA). Then, 10 µg of OVA adsorbed to Al(OH)_3_ was injected i.p. on days 0 and 7. Mice were challenged by an OVA-aerosol in saline on days 14 and 16. Mononuclear alveolar phagocytes were collected and enriched from broncho-alveolar lavage fluid on day 17.

### 4.7. Broncho-Alveolar Lavage (BAL) Fluid Collection and Culturing of Mononuclear Cells

OVA mice (*n* = 5), LPS mice (*n* = 5) and naïve mice (3-month-old BALB/c mice, *n* = 5) were anesthetized with an overdose of ketamine/xylazine. Thorax was opened, trachea exposed and a tracheal cannula (1.20 × 40mm needle) inserted and fixed with a thread. The lung was lavaged 8 times with 1 mL ice-cold BAL buffer (PBS with 0.6 mM EDTA) and collected in a Falcon tube on ice. Erythrocytes were lysed with 5 mL of NH_4_Cl_2_ lysis buffer (9 g NH_4_Cl, 1 g KHCO_3_, 37 mg EDTA in 100 mL Fresenius water) on ice. Cells were washed and the number of viable cells was evaluated with Trypan blue. Cells were resuspended in monocyte adherence medium (same as for human PBMC) to get a final concentration of 0.3 million mononuclear cells/mL. Subsequently, 150,000 mononuclear cells from LPS and OVA or 75,000 from naïve mice per well were seeded in a 48-well plate (Corning^®^ CellBIND, Sigma-Aldrich, St. Louis, Missouri, USA). After 1.5 h incubation at 37 °C, non-adherent cells were washed off (3 × 500 µL PBS) and the medium was replaced by RPMI medium supplemented with 10% FCS and 1% penicillin/streptomycin. Cells were cultured at 0.15 million mononuclear cells/mL medium and conditioned medium was collected after 4 and 18 h.

### 4.8. Leukocyte Sorting from Whole Lung Single-Cell Suspension

Mice were anesthetized with an overdose of ketamine/xylazine before whole lungs were excised, inflated with 10 U/mL Dispase II (#04942078001, Roche, Basel, Switzerland) solution and the trachea was closed by instillation of 500 µL of pre-warmed 1% low-melt agarose solution (#A0701-25G, Sigma-Aldrich). Subsequently, lungs were placed into 50 mL Falcon tubes containing 4 mL of Dispase II solution and incubated for 40 min at room temperature. Lung lobes were separated and dissociated with sterile scissors in DMEM media supplemented with DNase I (#LS002006, Worthington, Columbus, Ohio, USA) followed by 8–10 min on an orbital shaker. The remaining tissue was gently loosened by pipetting, the solution was passed through a 100 µm cell strainer and washed with DMEM supplemented with 10% FCS. Red blood cells were lysed with NH_4_Cl_2_ buffer for 3 min on ice. Lysis was stopped with PBS and the cell suspension was passed through a 40 µm cell strainer. Total cell number and viability were obtained with Trypan blue. An average of 20–30 million cells were stained for each mouse, first with viability dye (1:1000 in PBS, Zombie aqua, BioLegend, San Diego, USA) for 20 min, washed with 300 µL PBS and spun down. Unspecific binding was blocked by incubation with mouse FcX block (1:100 in PBS with 2% FCS, BioLegend) for 15 min at 4 °C. Cell populations were stained in 200 µL antibody cocktail (as listed in Table S1) for 30 min at 4 °C, spun down and resuspended in complete medium (RPMI with 2.5% FCS and 1% penicillin/streptomycin) at a cell density of 16 million/mL. Monocytes (CD11b^+^, Ly6G^-^, Ly6C^+^), macrophages (CD11b^+^, Ly6G^-^, Ly6C^-^, F4/80^+^) and mast cells (CD11b^-^, c-kit^+^) were sorted from viable CD45^+^ cells with a FACSAria device (BD Biosciences). For the determination of lipid mediator content in conditioned medium with LC/MS, cells obtained from one whole lung were split in half and resuspended in culture medium (300 µL RPMI medium supplemented with 2.5% FCS and 1% penicillin/streptomycin, 48-well plate) with or without 50 ng/mL LPS and 10 ng/mL IFN-γ directly after sorting and incubated overnight (18 h) in a humidified atmosphere with 5% CO_2_ at 37 °C. A small aliquot of sorted cell populations was spun down using a Cytospin3 device (Shandon, Marshall Scientific, Hampton, USA; 600 rpm for 3 min) and stained with a standard Diff/Quik protocol (Hemacolor, Sigma-Aldrich, St. Louis, Missouri, USA) for morphological characterization.

### 4.9. Flow Cytometry

For samples stained with more than one fluorophore-conjugated antibody, a cell-based compensation was performed with FACS Diva Software and adapted, if necessary, with FlowJo Diagnostic Software version 10. A list of antibodies can be found in Table S2.

#### 4.9.1. hPGDS Staining of Human Leukocytes

To differentiate between immune cell subsets, 3 million PBMC or PMNL per tube were stained for 15 min at room temperature with cell-specific surface markers and populations were characterized as indicated: monocytes (CD14^+^CD16^+^), CD4^+^ T-cells (CD3^+^CD4^+^), CD8^+^ T-cells (CD3^+^CD8^+^), natural killer cells (CD3^-^CD56^+^), NK/T cells (CD3^+^CD56^+^), B-cells (CD19^+^CD20^+^), plasma cells (CD138^+^), dendritic cells (HLA-DR^+^CD123^+^), basophils (HLA-DR^-^CD123^+^), neutrophils (PMNL, CD16^+^), eosinophils (PMNL, CD16^-^). Monocyte-derived macrophages were collected as described earlier, pre-fixed for 30 min in fixation buffer and used for hPGDS staining. Mast cells, and in parallel CD14^+^ monocytes, were stained in whole blood. Erythrocytes in 1 mL whole blood were lysed by incubation with NH_4_Cl_2_ buffer for 10 min on ice and lysis was stopped by adding excess PBS. Cells were stained with FITC-conjugated anti-c-kit (#313231, 1:20, BioLegend), PE-conjugated anti-Fcε-RI (#334609, 1:20, BioLegend) and BV421-conjugated anti-CD14 (#367143, 1:100, BioLegend) for 20 min at room temperature. Cells were prepared for intracellular staining with a Fixation/Permeabilization Kit I (BD Biosciences). Unspecific binding was reduced by incubation with a 1:1 mixture of UV-block (BD Bioscience) and 5% FCS in PBS or human FcX-receptor block (1:100, BioLegend) for 30 min. Subsequently, cells were stained with primary monoclonal mouse anti-human hPGDS antibody (1:100, MAB6487, Novus Bioscience) or the corresponding isotype control (mouse IgG, Novus Bioscience) in permeabilization wash buffer as recommended for 30 min, followed by incubation with secondary antibody AF488-conjugated rabbit anti-mouse IgG (1:5000, ThermoFisher Scientific) or AF647-conjugated goat anti-mouse IgG (whole blood; 1:500, ThermoFisher Scientific) for 30 min.

#### 4.9.2. hPGDS Staining of Murine Leukocytes

After population staining as described for sorting, a fraction of the whole lung single-cell suspension was divided into FACS tubes (~ 250 000 cells/tube) for markers only (FMO), 2^nd^ only, and hPGDS staining. Cells were fixed and permeabilized according to the manual using Fixation/Permeabilization Kit I from BD Bioscience and with unspecific binding blocked by incubation with 50 µL of 1:100 dilution of mouse FcX block for 30 min. Subsequently, cells were stained with 50 µL of primary rabbit anti-hPGDS (1:100 in perm/wash buffer, LS-B6886, LS Bio, Seattle, Washington, USA) for 30 min at 4 °C. After washing, 100 µL Pacific blue-conjugated goat anti-rabbit secondary antibody (ThermoFisher Scientific, 1:500) was added for 30 min at 4 °C. hPGDS expression was determined with FACS Canto II and FlowJo Software by calculation of fold increase over FMO.

### 4.10. hPGDS Immunostaining for Microscopy

A total of 500 000 human peripheral blood monocytes/well, isolated with a Monocyte Isolation Kit II (Miltenyi), were seeded into Poly-L-Lysine pre-coated Lab-Tek II CC^2^ 8-well chamber slides (ThermoFisher Scientific) and left to adhere for 4 h. For MDM staining, 250 000 monocytes/well were seeded into Poly-L-Lysine pre-coated 8-well chamber slides and differentiated into MDM as described above. Cells were fixed with 3.8% formalin solution for 15 min and permeabilized with 0.1% TritonX-100 in phosphate-buffered saline for 15 min. Subsequently, cells were stained with primary mouse anti-human hPGDS (1:100, MAB6487, Novus Bioscience, Colorado, USA) for 1 h at room temperature and subsequently with secondary AF488-conjugated goat anti-mouse antibody (1:500, ThermoFisher Scientific) and TexasRed-conjugated phalloidin (ThermoFisher Scientific) for 30 min at room temperature in the dark. Slides were mounted with a DAPI fluorescence mounting medium (Vectashield, Burlingame, CA, USA) and images were acquired with a NIKON A1 confocal microscope at 60x magnification. Controls included unstained cells, cells stained with isotype and secondary antibody only.

#### Human Precision-Cut Lung Slices

Formalin-fixed, paraffin-embedded PCLS sections (4 µm) were deparaffinized and subjected to heat-induced antigen retrieval in sodium citrate buffer at pH 6, followed by a quenching step (glycin 1 mg/mL in a.d. for 30 min, then 1 mg/mL sodium borohydrate in PBS for 3 × 10 min). Unspecific binding was blocked with 4% BSA and 10% goat serum in PBS for 2 h at room temperature. Primary antibodies (listed in the Supplementary Material, Table S2) were applied diluted 1:100 in blocking solution diluted 1:10 with PBS overnight at 4 °C. After washing steps, sections were incubated with donkey anti-rabbit and goat anti-mouse immunoglobulin labelled with Alexa Fluor dyes 647 or 488, respectively (both from ThermoFisher Scientific, 1:500) for 2 h. Nuclear counterstaining was performed with DAPI mounting medium (Vector Laboratories, Burlingame, CA, USA). Images were taken with a Nikon A1 confocal microscope (Nikon Inc., Tokyo, Japan).

### 4.11. In Situ Hybridization

ISH was conducted as described previously [51] with the RNAscope kit (Cat#322360, ACDBio, Newark, CA, USA) for hPGDS (RNAscope Probe Mm-Hpgds, ACDBio, Newark, CA, USA, Cat#413391) according to the manufacturer’s protocol. Briefly, lung slides were deparaffinized with xylene and 99.9% EtOH followed by drying in a pre-heated oven. Subsequently, slides were transferred to a steamer containing MilliQ water for acclimatization before antigen retrieval with 10 mM citrate buffer pH 6 was performed by microwaving at 850 W for 10 min. Slides were rinsed with MilliQ water and equilibrated with 99.9% EtOH for 3 min before being dried in an oven at 37 °C. Each section was treated with protease plus (RNAScope kit), covered and incubated in the oven humidity tray at 40 °C for 20 min. Again, slides were rinsed with MilliQ water, placed back onto the slide rack and a probe was added (RNAscope Probe- Mm-Hpgds, Cat#413391). The rack was placed back into the oven and probe hybridization was performed at 40 °C for 2 h. RNAscope wash buffer was diluted with MilliQ water and pre-warmed to 37 °C. Slides were transferred one by one to wash buffer and incubated for 2 min at room temperature with occasional agitation. This step was repeated once. Next, slides were incubated with amplifier 1 for 30 min, amplifier 2 for 15 min, amplifier 3 for 30 min, amplifier 4 for 15 min, amplifier 5 for 45 min and amplifier 6 for 15 min. Amplification steps 1 to 4 were performed at 40 °C, while steps 5–6 were performed at room temperature. In between amplification steps, slides were washed twice in wash buffer for 2 min each. Finally, sections were incubated with detection reagent (Fast Red-A to Fast Red-B in a 1:60 ratio, 60 µL per section) in the humidity control tray for 10 min at room temperature. Slides were washed in MilliQ water, mounted with a DAPI-containing Vectamount medium and imaged with a NIKON A1 confocal microscope at 20× magnification.

### 4.12. Real-Time Quantitative PCR

A reverse transcription control was performed by mixing 1 µL RNA from randomly chosen samples, adding the reaction mix but no reverse transcriptase. Additionally, a negative control (nuclease-free water) were run in parallel during each qPCR run (2 min−95 °C, 39 to 55 repeats of 5 s −95 °C and 30 s of 60 °C, melting curve: 5 s−95 °C, 5 s−65 °C, 0.5 °C increments). DeltaCq (ΔCq) values were calculated by subtracting corresponding gene Cq from housekeeper (GAPDH or β-actin, as indicated) Cq values. Some results were plotted as 2^ΔCq^ (normalization to housekeeping gene) or 2 ^ΔΔCq^ (additional normalization to control) values. N number indicates biological replicates from at least two technical replicates.

#### 4.12.1. Human Leukocytes

Fully differentiated MDM were collected in TriReagent at indicated time points after activation and stored at −70 °C until further use. Additionally, monocytes isolated with the Monocyte Isolation Kit II were used for RNA extraction and RT-qPCR (0.5 to 1 million cells per condition). Phase separation was achieved with chloroform and centrifugation at maximum speed, while RNA precipitation was induced with molecular-grade ethanol. According to the protocol, samples were further purified with an RNeasy Mini Kit (Qiagen, Hilden, GER) and RNA was eluted in nuclease-free water. 0.3 to 1 μg of RNA were reverse transcribed using iScript cDNA Synthesis Kit (Bio-Rad, Herkules, CA, USA). HPGDS, COX-2, mPTGES-1 and GAPDH gene expression was detected using a combination of SsoAdvanced^TM^ Universal SYBR^®^ Green Supermix (Bio-Rad, Herkules, CA, USA) with validated PrimePCR^TM^ SYBR^®^ Green Assay primers.

#### 4.12.2. Murine Leukocytes 

Briefly, sorted macrophage, monocyte and mast cell samples from vehicle and LPS mice were thawed and processed simultaneously. Phase separation was achieved by the addition of chloroform and centrifugation as described above. RNA was precipitated by the addition of the same volume of 2-propanol and incubation for 5 min at room temperature. Pellet was washed twice with 75% molecular grade EtOH and air-dried. RNA was resuspended in 11 µL nuclease-free water. A total of 5 µL of RNA solution from each sample was reverse transcribed using the High-capacity cDNA kit (ThermoFisher Scientific) and the resulting cDNA was diluted with 25 µL nuclease-free water. Then, 3 µL of sample cDNA was assayed in a 10 µL PCR reaction (SsoAdvanced^TM^ Universal SYBR^®^ Green Supermix, BioRad) along with primer pairs listed in Table S3. Linearity of primer pair response was previously confirmed using a sample from LPS stimulated murine liver single-cell suspension (data not shown), which was also run parallel to each PCR reaction as a positive control.

#### 4.12.3. PCLS

Snap-frozen PCLS were homogenized in TriReagent by mechanical disruption followed by sonication (4 cycles at 40% power for 10 s each). RNA was extracted and cDNA generated (HighCapacity cDNA Kit, ThermoFisher Scientific) as described above. A total of 3 µL of sample cDNA was assayed in a 10 µL PCR reaction (SsoAdvanced^TM^ Universal SYBR^®^ Green Supermix, BioRad) along with primer pairs listed in Table S3.

### 4.13. Western Blotting

Monocyte and macrophage lysates were collected in protein lysis buffer (10 mM Hepes, 1 mM EDTA, 1% Triton, 1 mM sodium-orthovanadate, 7.5 µL protease inhibitor cocktail (Sigma)). Lysates were sonicated (4 cycles at 40% power for 10 s each), spun down at 12 000 rpm for 10 min at 4 °C and the supernatant was used for Western blotting. Protein from PCLS was extracted from TriReagent lysates after removal of the RNA layer. Briefly, the protein was precipitated with acetate overnight at −20 °C, collected by centrifugation and resuspended in 200 µL RIPA buffer (ThermoFisher Scientific) with HALT^TM^ protease and phosphatase inhibitor cocktail (ThermoFisher Scientific), 1% SDS, 50 mM Tris, 4M Urea, pH 8,0. Between 15 to 30 µg protein was loaded onto a precast gel (Novex WedgeWell, 4–20% Tris-Glycine mini Gels, ThermoFisher Scientific) and proteins separated with 225 V for 45 min. Protein bands were transferred onto a PVDF membrane with an iBlot gel transfer device (ThermoFisher Scientific). Unspecific binding was blocked with 5% milk in TBST buffer for 1 h and, subsequently, the membrane was incubated with primary rabbit anti-human hPGDS (LS-B6886, 1 µg/mL, LSBio), rabbit anti-human COX-2 (15191, 1:1000, Abcam, Cambridge, UK), rabbit anti-LPGDS antibody (1 µg/mL, NBP1-79280, Novus Biologicals) in 1% milk in washing buffer or rabbit anti-human GAPDH (2118S, 1:3000, New England Biolabs, Massachusetts, USA) in 5% milk at 4 °C overnight. The next day, the blot was incubated with a horseradish peroxidase-conjugated secondary antibody (1:5000 in 5% milk, Jackson Immuno) for 1.5 h. Bands were visualized by incubation for 5 min with Clarity™ Western ECL Blotting Substrate (BioRad) and subsequently evaluated with a BioRad chemiluminescence detector and ImageLab Software. If required, the blot was washed and incubated with stripping buffer (65.2 mM Tris/HCl with 2% SDS pH 6.9 with 100 nM β-mercaptoethanol) at 50 °C with shaking, washed and blocked for 1 h with 5% milk before incubation with additional primary antibody.

### 4.14. Prostaglandin D_2_-MOX ELISA

PGD_2_-MOX ELISA (CaymanChemicals, Ann Arbor, Michigan, USA) was performed according to the manufacturer’s protocol. The standards and the samples were diluted in a 1:1 mixture of culture medium and methyl-oximating reagent and assayed at adequate dilutions and in duplicates, where possible. In some experiments, obtained values were normalized to the total protein content per well as determined with a Pierce^TM^ BCA protein assay (ThermoFisher Scientific) or tissue wet weight (PCLS).

### 4.15. Prostaglandin E_2_ Radioimmunoassay (RIA)

Detection of immunoreactive PGE_2_ in conditioned medium was performed as described previously [52]. Briefly, samples and standards (Sigma) were incubated with PGE_2_ antiserum and [5,6,8,11,12,14,15(N)-3H]-PGE_2_ (PerkinElmer, Germany) overnight at 4 °C. Unbound PGE_2_ was removed by adding activated charcoal the next day, followed by centrifugation at 4000 rpm for 15 min at 4 °C. Subsequently, supernatants were poured into scintillation tubes and a scintillation cocktail (PerkinElmer) was added. Counts per minute were measured in a beta counter (HIDEX, Turku, Finland) and values were calculated in Sigma plot by employing a 4-parameter curve fit algorithm. In some experiments, obtained values were normalized to the total protein content per well as determined with a Pierce^TM^ BCA protein assay (ThermoFisher Scientific) or tissue wet weight (PCLS).

### 4.16. Determination of Lipid Mediators in Conditioned Medium with Liquid Chromatography and Mass Spectrometry (LC/MS)

This analysis was performed at the Pharmazentrum Frankfurt/ZAFES, Institute of Clinical Pharmacology, Goethe University Frankfurt, Germany. Briefly, for the analysis of prostanoids in cell supernatants, 230–285 µL of the samples (on ice water) was spiked with 20 µL of a solution of the isotopically labeled internal standards in ethanol (PGE_2_-d4, PGD_2_-d4, TXB_2_-d4: 10 ng/mL). After the addition of 20 µL pure ethanol and 100 µL of an aqueous EDTA solution (0.15 M), the sample was extracted twice using liquid–liquid extraction. For the extraction, 600 µL of ethyl acetate was added and after mixing (1 min) and centrifugation (3 min at 20,000× *g*, 4 °C) the supernatant was collected. The extraction was repeated and the supernatants were joined together before evaporation at 45 °C under a nitrogen stream. After reconstitution in 50 µL water:acetonitrile (8:2, v/v) containing 0.0025% formic acid, samples were mixed, centrifuged (1 min at 20,000× *g*) and transferred into vial inserts. The chromatographic separation was carried out using an Acquity UPLC BEH C18 column (2.1 × 100 mm, 1.7 µm, Waters Corporation, Milford, Massachusetts, USA) under gradient conditions at 30 °C. Water and acetonitrile, both containing 0.0025% formic acid, were used as mobile phases and the run time was 13 min. The LC-MS/MS system consisted of a 1290 Infinity II HPLC pump and autosampler (G7120A + G7167B, Agilent Technologies, Santa Clara, California, USA) and of a hybrid triple quadrupole-ion trap mass spectrometer QTrap 6500+ (Sciex, Darmstadt, Germany) equipped with a Turbo-V-source operating in negative ESI mode. Analysis was done in MRM mode with a dwell time of 10 ms for all analytes. The injection volume was 10 µL. Data were acquired using Analyst Software V1.7.1 and quantified with MultiQuant Software V 3.0.3 (both Sciex, Darmstadt, Germany), using the internal standard method (isotope dilution mass spectrometry).

### 4.17. LEGENDplex^TM^ Multiplex ELISA

LEGENDplex^TM^ Human Inflammation Panel 1 (13-plex) kit (BioLegend) was performed according to the manufacturer’s protocol. Briefly, 12.5 µL of undiluted PCLS conditioned media was assayed, recorded with a FACSCalibur (BD Biosciences) and analyzed with the provided Data Analysis Software Suite (BioLegend, version 8) to determine respective cytokine concentrations. Obtained values were normalized to tissue wet weight.

### 4.18. Statistical Analysis

All experiments show data from at least two technical replicates, while the *n* number reflects biological replicates. Data are shown as mean ± SEM or box-and-whisker plots with boxes representing the quartiles and whiskers indicating the range. Comparisons of groups were performed as appropriate with GraphPad Prism 9 software (San Diego, CA, USA). Residuals from each analysis were tested for normality using a Shapiro–Wilks test (α = 0.05). Datasets that did not pass the normality test were log-transformed, re-evaluated for normal distribution (Shapiro–Wilks test) and when normality could be confirmed statistical differences were calculated with a parametric test using the log-transformed values. Datasets that did not pass the log-normality test were analyzed with a non-parametric test (Kruskal–Wallis). Homogeneity of variance was tested and corrected for using the Brown–Forsythe test in unmatched datasets and the Geisser–Greenhouse correction in matched datasets. Probability values of *p* < 0.05 were considered statistically significant. Specific tests used for each dataset are indicated in the figure legend.

## Data Availability

The data presented in this study are included in the article and supplementary material. Requests of different nature can also be made to the corresponding authors on a reasonable basis.

## Supplementary Materials

Available online at www.mdpi.com/1422-0067/222/11/1697/s1.
