# Supplementary material for: Monocytes and Macrophages Serve as Potent Prostaglandin D2 Sources during Acute, Non-Allergic Pulmonary Inflammation
Source: Int J Mol Sci. 2021 Oct 28;22(21):11697. doi: 10.3390/ijms222111697 (PMC8584273; doi:10.3390/ijms222111697)
Supplement: Supplementary file 1 [file ijms-22-11697-s001.zip › ijms-1422362-supplementary.pdf]

Supplementary material - Monocytes and macrophages serve as potent Prostaglandin D_2 sources during acute, non-allergic pulmonary inflammation

Tables:

Table S1. Myeloid panel for murine lung single cell suspension.

	Company	Category #	Dilution
**FITC anti-mouse Ly6C**	BioLegend	128005	1:600
**APC anti-mouse Ly6G**	BioLegend	127613	1:400
**PE-Cy7 anti-mouse CD11b**	BioLegend	561098	1:200
**PerCP anti-mouse CD45**	BioLegend	103129	1:200
**PE anti-mouse F4/80**	BioLegend	565410	1:50
**APC-Cy7 anti-mouse c-kit**	BioLegend	105825	1:100
**Zombie aqua viability dye**	BioLegend	423101	1:500

Table S2. List of antibodies.

Antibodies for Flow cytometry	Company	Cat.#	dilution
**APC mouse anti-human CD123**	BD Bioscience	560087	1:5
**PE Isotype Control IgG1 kappa**	BD Bioscience	559320	1:5
**PE mouse anti-human CD138**	BD Bioscience	561704	1:5
**PE mouse anti-human CD206**	BD Bioscience	555954	1:5
**PE mouse anti-human CD4**	BD Pharmingen	561843	1:5
**PE mouse anti-human CD8**	BD Pharmingen	560949	1:5
**PE mouse anti-human CD80**	BD Bioscience	557227	1:5
**PE mouse anti-human HLA/DR**	BD Bioscience	556653	1:5
**PE-Cy5 mouse anti-human CD16**	BioLegend	302010	1:20
**PE-Cy5.5 mouse anti-human CD3**	BD Bioscience	555334	1:5
**PE-Cy7 mouse anti-human CD20**	BD Pharmingen	560735	1:20
**PE mouse anti-human CD56**	BD Pharmingen	5555516	1:5
**PerCP mouse anti-human CD14**	BD Bioscience	2240746	1:50
**BV421 mouse anti-human CD19**	BD Horizon	560354	1:20
**Primary antibodies**			

**Mouse anti-hPGDS (IgG1)**	Novus Bioscience	MAB6487	1:100
**Mouse IgG1 isotype control**	Novus Bioscience	MAB002	1:100
**Rabbit anti-hPGDS**	LSBio	LS-B6886	1:100
**Rabbit anti-human GAPDH**	New England Biolabs	2118S	1:5000
**Mouse anti-human Mast Cell Tryptase**	LSBio	LS-B2347	1:400
**Mouse anti-human CD68**	Abcam	ab955	1:100
**HRP-conjugated antibodies**			
**goat anti-rabbit HRP**	Jackson ImmunoResearch	111-035-045	1:5000
**goat anti-mouse HRP**	Jackson ImmunoResearch	115-035-062	1:5000
**Alexa Fluor-conjugated secondary antibodies**			
**AF488 goat anti-mouse**	ThermoFisher	A-10667	1:500
**AF Pacific blue goat anti-rabbit**	ThermoFisher	P-10994	1:500
**AF647 donkey anti-rabbit**	ThermoFisher	A-31573	1:500
**Others**			
**TexasRed-X Phalloidin**	ThermoFisher	T7471	1:40
**TruStain FcX (anti-mouse CD16/32)**	BioLegend	101320	1:20
**TruStain FcX (human Fc receptor)**	BioLegend	422302	1:100
**Zombie aqua viability dye**	BioLegend	423101	1:1000

Table S3. List of primers.

Gene	species	NCBI ID	primerbank ID / origin	Forward primer	Reverse primer
**HPRT1**	ms	15452		AGGCCAGACTTTGTTGGATTTGAA	CAACTTGCGCTCATCTTAGGCTTT
**HPGDS**	ms	54486	254281299c1	AAGCTGACTGGCCTAAAATCAAG	CTCTGGTGGATTGTAAGTCCTTC
**mPGES1**	ms	64292	11967941a1	GGATGCGCTGAAACGTGGA	CAGGAATGAGTACACGAAGCC
**COX-2**	ms	19225	31981525a1	TGAGCAACTATTCCAAACCAGC	GCACGTAGTCTTCGATCACTATC
**LPGDS**	ms	19215	2317286a1	TGCAGCCCAACTTTCAACAAG	TGGTCTCACACTGGTTTTTCCT
**β-actin**	h	60	PMC4037220	CCTCACCCTGAAGTACCCCA	TGCCAGATTTTCTCCATGTCG
**COX-2**	h	5743	PMC4037220	GAATCATTCACCAGGCAAATTG	TTTCTGTACTGCGGGTGGAAC
**mPGES1**	h	9536	PMC4037220	CTGCTGGTCATCAAGATGTACG	GGTTAGGACCCAGAAAGGAGT
**HPGDS**	h	27306	Sigma-Aldrich	ACCAGAGCCTAGCAATAGCAA	AGAGTGTCCACAATAGCATCAAC
**TNF-α**	h	7124	Origene	CTCTTCTGCCTGCTGCACTTTG	ATGGGCTACAGGCTTGTCACTC
**IL-6**	h	3569	Eurofins	GTAGCCGCCCCACACAGA	CATGTCTCCTTTCTCAGGGCTG

Supplementary Figures:

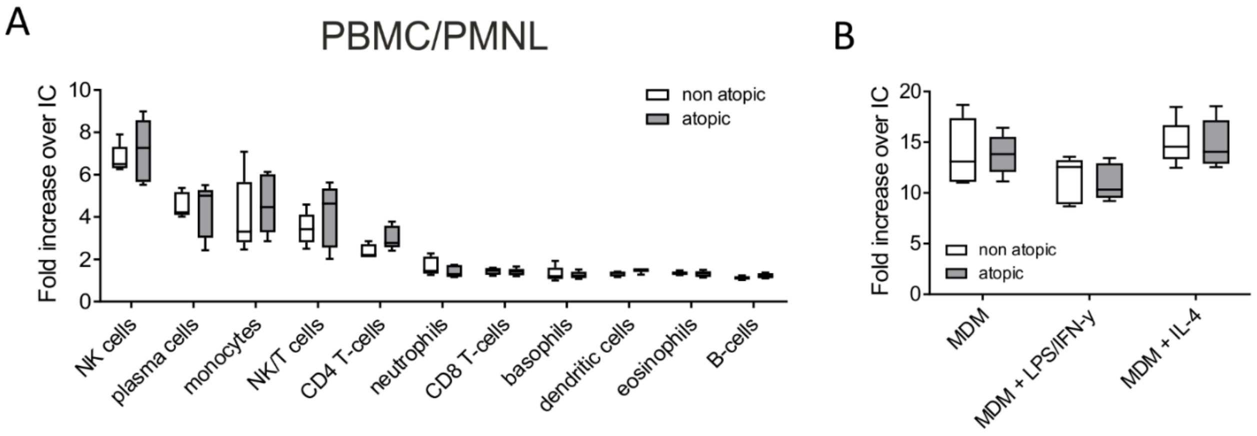

Figure S1. Similar levels of hPGDS expression in leukocytes isolated from atopic or non-atopic donors. hPGDS expression was evaluated (A) in the PBMC and PMNL fraction of peripheral blood or (B) monocyte-derived macrophages from non-atopic and atopic donors. MDM were either left unstimulated or polarized with LPS/INF-γ or IL-4 for 48 h. No significant difference could be observed. n = 5.

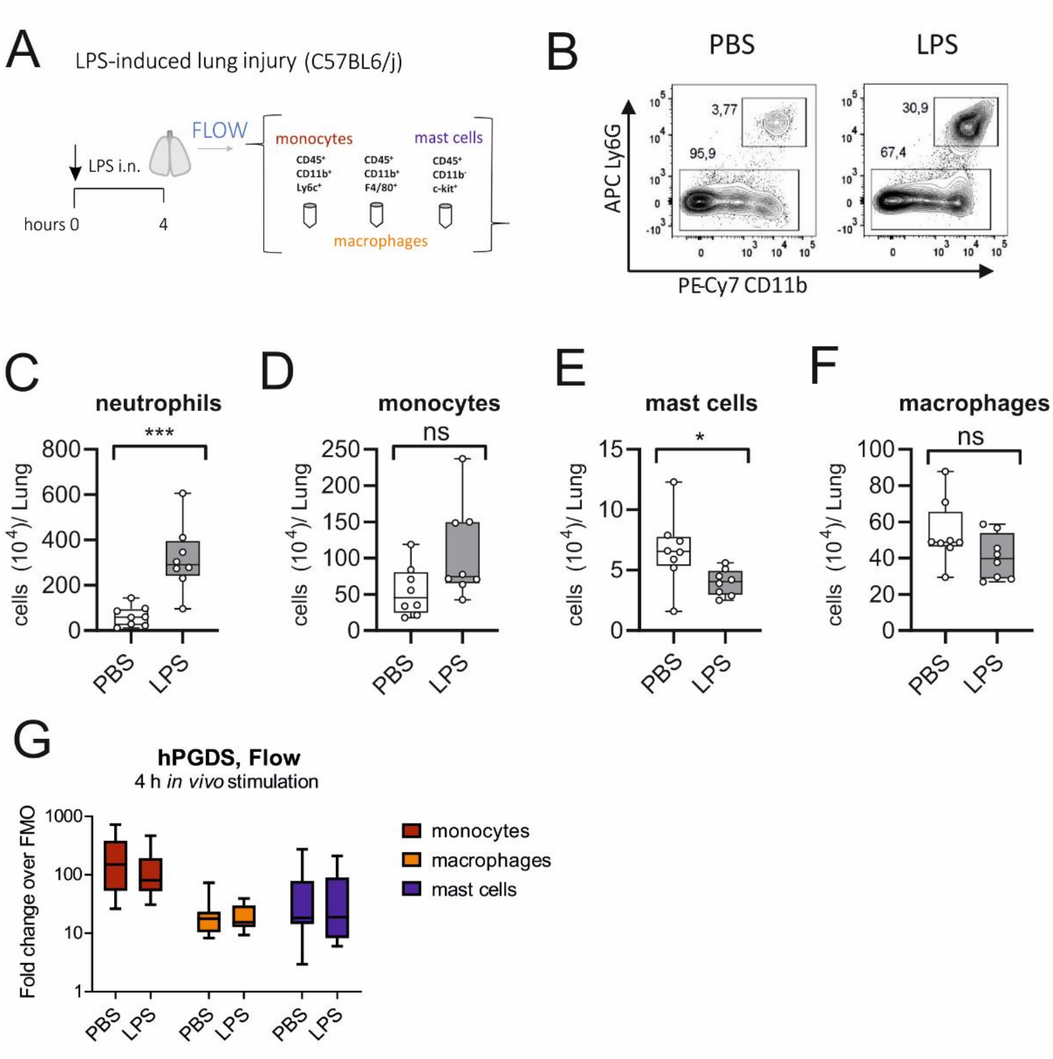

Figure S2. Numbers of pulmonary neutrophils, monocytes, macrophages and mast cells from mice with LPS-induced pulmonary inflammation and controls. (A) Experimental setup. (B) and (C) 4 h after intranasal LPS, a significant neutrophilic influx could be confirmed. (D) Monocyte numbers were increased by trend, (E) while mast cell numbers were significantly reduced. (F) Macrophages were found in slightly lower numbers in LPS lungs at this timepoint. (G) LPS-induced lung inflammation for 4 h did not result in increased hPGDS expression. Data are shown as box-and-whisker plots with one data point representing one mouse, n = 8, Student's t-test, * $p < 0.05$.

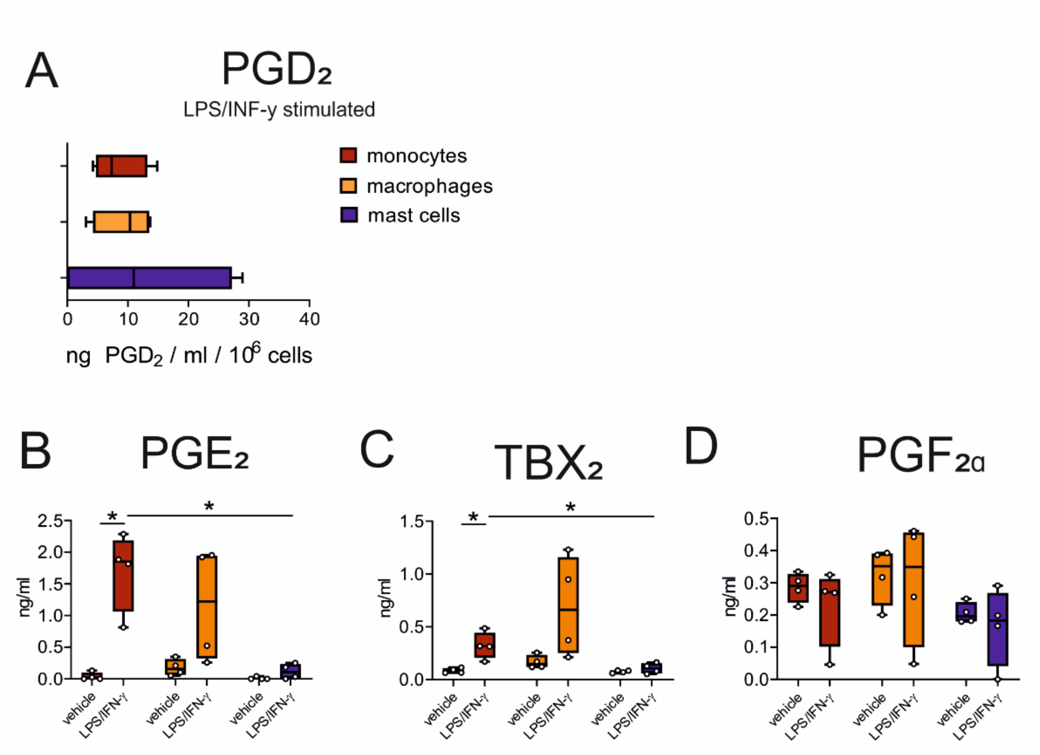

Figure S3. Prostanoid release after *in vitro* stimulation of pulmonary monocytes, macrophages and mast cells with LPS/IFN-γ. After sorting, populations were divided up and one half stimulated with vehicle, the other with LPS/IFN-y and incubated for 18 h. (A) Murine pulmonary monocytes and macrophages released PGD_2 in the ng range per million cells (calculated from data shown in Figure 2F). (B) Monocytes released large amounts of PGE_2, (C) TBX_2 was also significantly elevated, while (D) PGF_{2a} levels were unchanged. n = 4, two-way ANOVA for repeated measurements, * $p < 0.05$.

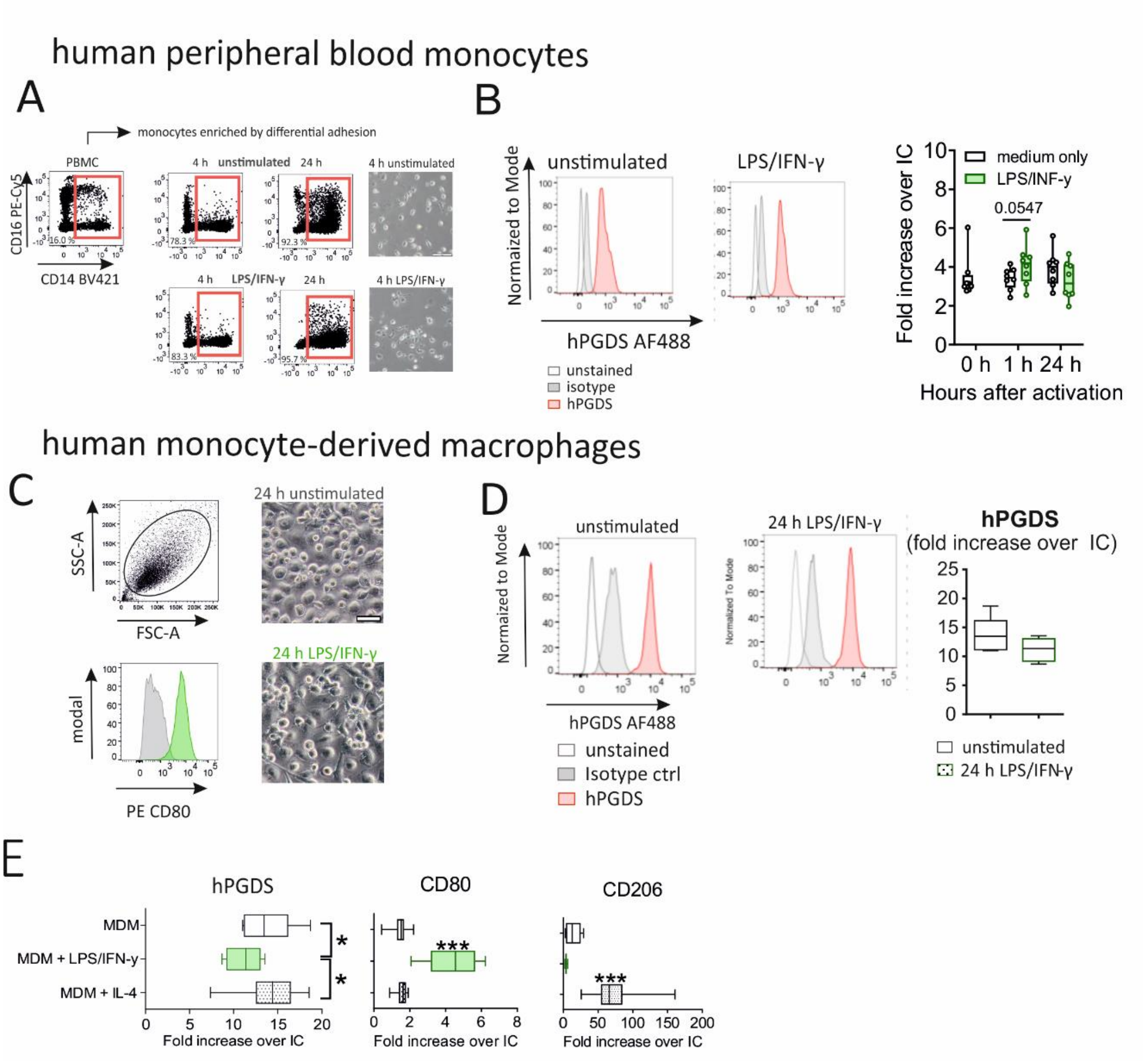

Figure S4. Characterization of human monocytes and monocyte-derived macrophages. (A) Peripheral blood monocytes in the PBMC fraction of human donors were enriched by differential adhesion and either left unstimulated or activated with LPS/IFN-γ. (B) hPGDS expression 1 h or 24 h after activation with LPS/IFN-γ was not significantly changed in monocytes. (C) To obtain monocyte-derived macrophages (MDM), peripheral blood monocytes were enriched as described above and differentiated with 20 ng/ml M-CSF for 6-8 days. MDM were either left unstimulated or actived with LPS/IFN-γ, which upregulated CD80 and changed their morphology. (D) hPGDS expression 24 h after stimulation was unchanged, while (E) MDM activated with LPS/IFN-y for 48 h expressed significantly less hPGDS. Successful activation was confirmed by evalulation of CD80 or CD206 expression. n = 5-10, One-way ANOVA for repeated measurements with Dunnett‘s *post hoc* test.

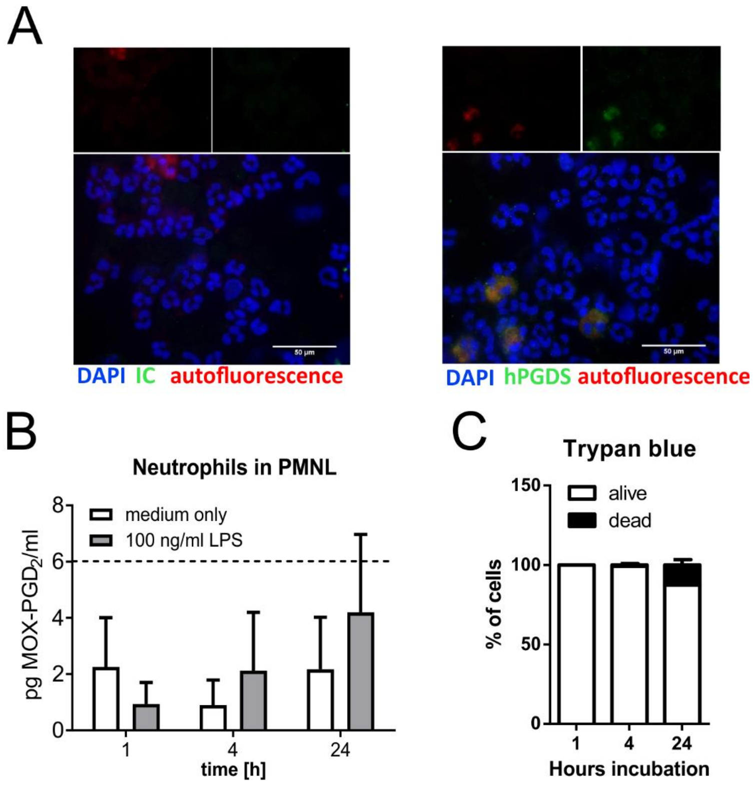

Figure S5. Human neutrophils express only low levels of hPGDS and don't release detectable amounts of PGD_2 after LPS stimulation *in vitro*. (A) Immunofluorescence staining with isotype control or hPGDS-specific antibody in PMNL fraction of heathy human donors. hPGDS-specific staining is shown in green; neutrophils were hardly stained. Autofluorescent eosinophils stained faintly positive for hPGDS. (n = 3, scale bar 50 µm) (B) LPS (100 ng/ml) stimulation of human neutrophils in the PMNL fraction of healthy donors did not result in PGD_2 levels above detection threshold (dotted line) (n = 4). (C) Viability of cells was determined with Trypan blue at the moment of conditioned medium collection (n = 4). Data are displayed as bar chart, mean + SEM.

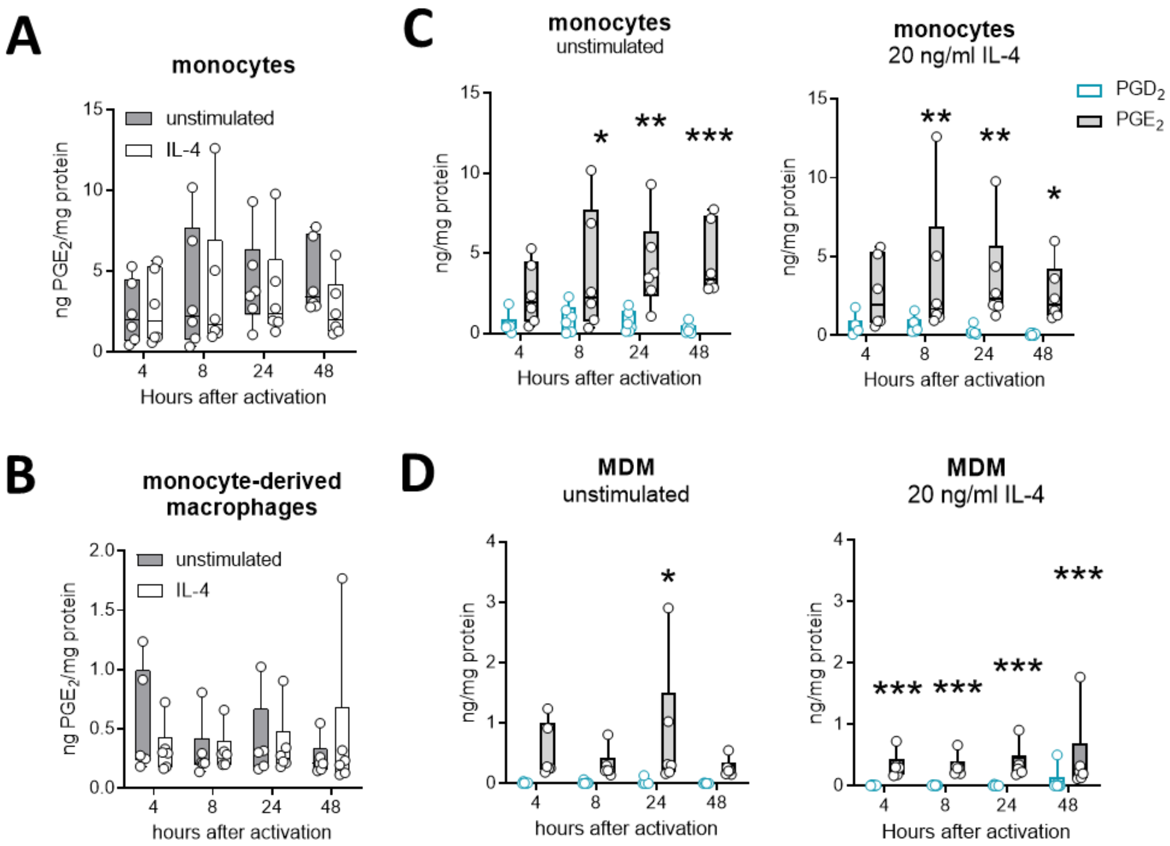

Figure S6. Human monocytes and monocyte-derived macrophages release low, but steady levels of PGE_2, while IL-4 does not have an influence. (A) Human peripheral blood monocytes and (B) monocyte-derived macrophages release up to 5 ng PGE_2/mg protein, but hardly any PGD_2, even when unstimulated. IL-4 did not increase PGE_2 release. Significantly higher levels of PGE_2 than PGD_2 could be measured in conditioned medium from (C) monocytes and (D) monocyte-derived macrophages stimulated with IL-4. Data are shown as box-and-whisker plot, n = 6, two-way ANOVA for repeated measurements with Sidak‘s *post hoc* test, ** $p<0.01$, *** $p<0.001$.

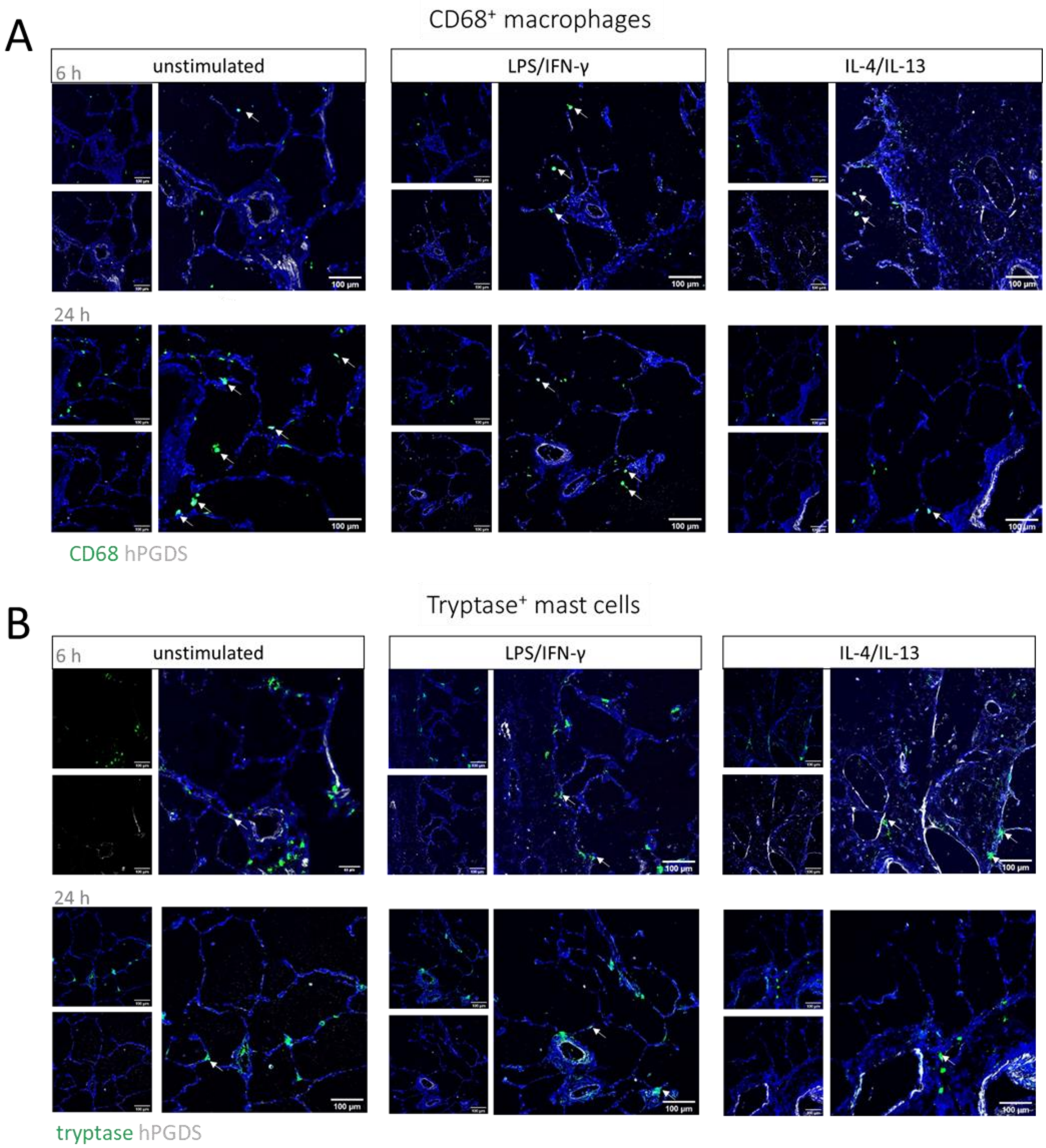

Figure S7. hPGDS expression in human pulmonary resident macrophages and mast cells after LPS/IFN-γ or IL-4/IL-13 stimulation. Serial cut precision cut lung slices from human donors were either left unstimulated or activated with LPS/IFN-γ or IL-4/IL-13 for indicated time. (A) Most $CD68^+$ cells stained highly positive for hPGDS, while staining intensity appeared increased after stimulation. (B) $Tryptase^+$ mast cells were present in similar numbers, though different location than alveolar macrophages and expressed low levels of hPGDS. Images are representative for 4 donors.

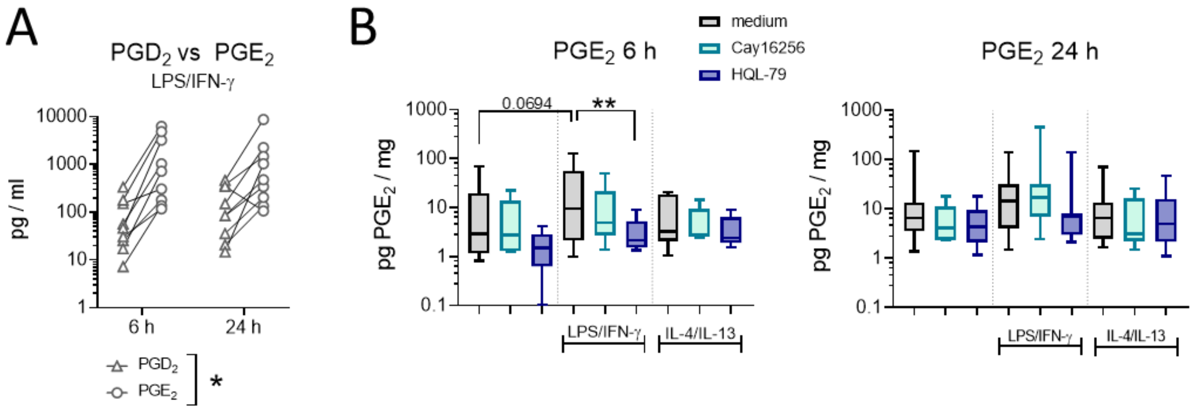

Figure S8. PGE$_2$ release by human donor PCLS after LPS/IFN-γ or IL-4/IL-13 stimulation. PGE$_2$ levels were measured in PCLS-conditioned medium to enable a comparison to PGD$_2$ levels. (A) Significantly higher amounts of PGE$_2$ than PGD$_2$ were present in PCLS-conditioned medium after LPS/IFN-γ stimulation. (B) PGE$_2$ increase after LPS/IFN-γ is less pronounced due to high levels released already by unstimulated PCLS. hPGDS inhibitor HQL-79 significantly reduced PGE$_2$ release at the 6 h time point. Data are shown as box-and-whisker or before-after plot, n = 5-9, One-way ANOVA for repeated measurements (mixed model) with Sidak's *post hoc* test, ** $p<0.01$.

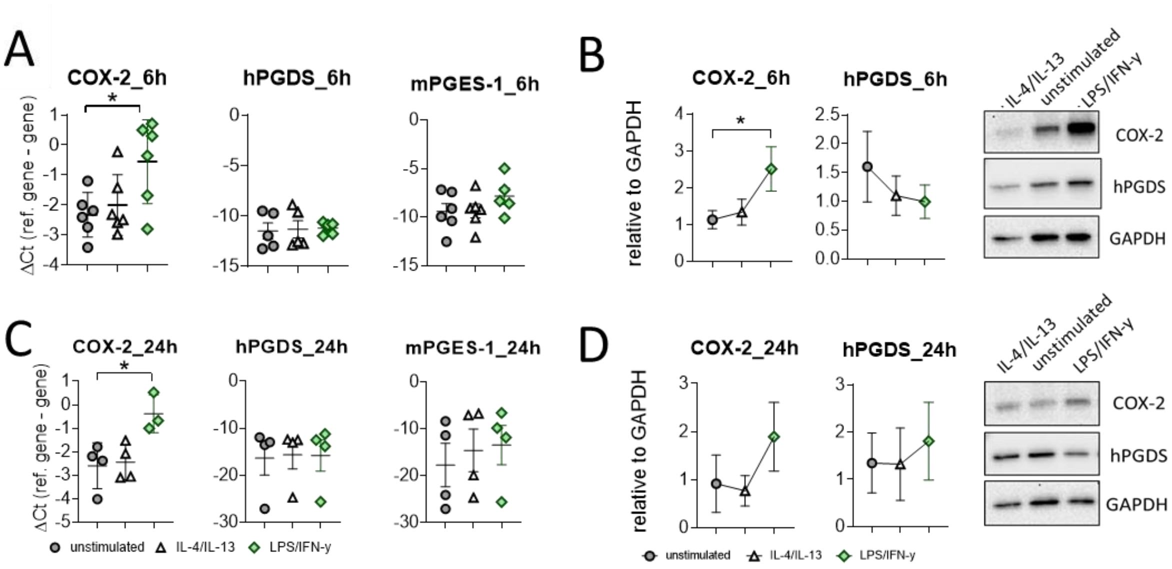

Figure S9. Regulation of COX-2, hPGDS, mPGES-1 after LPS/IFN-y and IL-4/IL-13 stimulation. (A) COX-2 mRNA and (B) protein was upregulated in PCLS activated with LPS/IFN-γ for 6 h. hPGDS and mPGES-1 were not significantly regulated. After 24 h stimulation, (C) COX-2 mRNA was still upregulated, (D) but less apparent on protein level. hPGDS expression was unchanged. Data are shown as line or scatter plots (mean ± SEM), n = 3 – 6. One-way ANOVA for repeated measurements (mixed model) with Dunnett's (A-D) or Sidak's (E, F) *post hoc* test, * $p<0.05$.

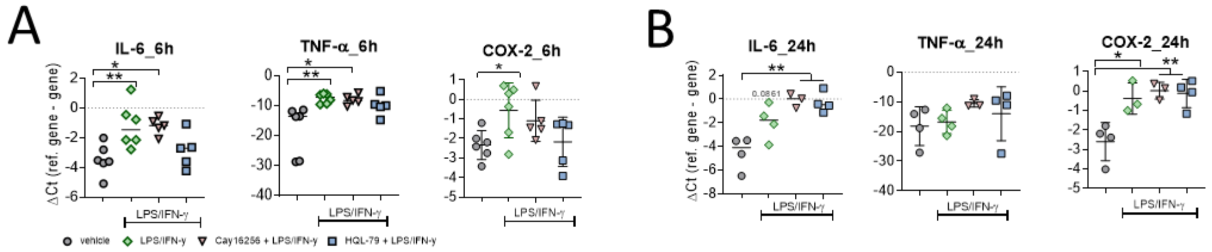

Figure S10. Regulation of IL-6, TNF-α and COX-2 mRNA levels after LPS/IFN-y stimulation in the presence of hPGDS inhibitors. Inhibition of hPGDS during stimulation with LPS/IFN-γ partly counteracted upregulation of (A) IL-6, TNF-α and COX-2 mRNA at 6 h, (B) but not at 24 h. Data are shown as line or scatter plots (mean ± SEM), n = 3 – 6. One-way ANOVA for repeated measurements (mixed model) with Dunnett‘s (A-D) or Sidak‘s (E, F) *post hoc* test, * $p<0.05$.

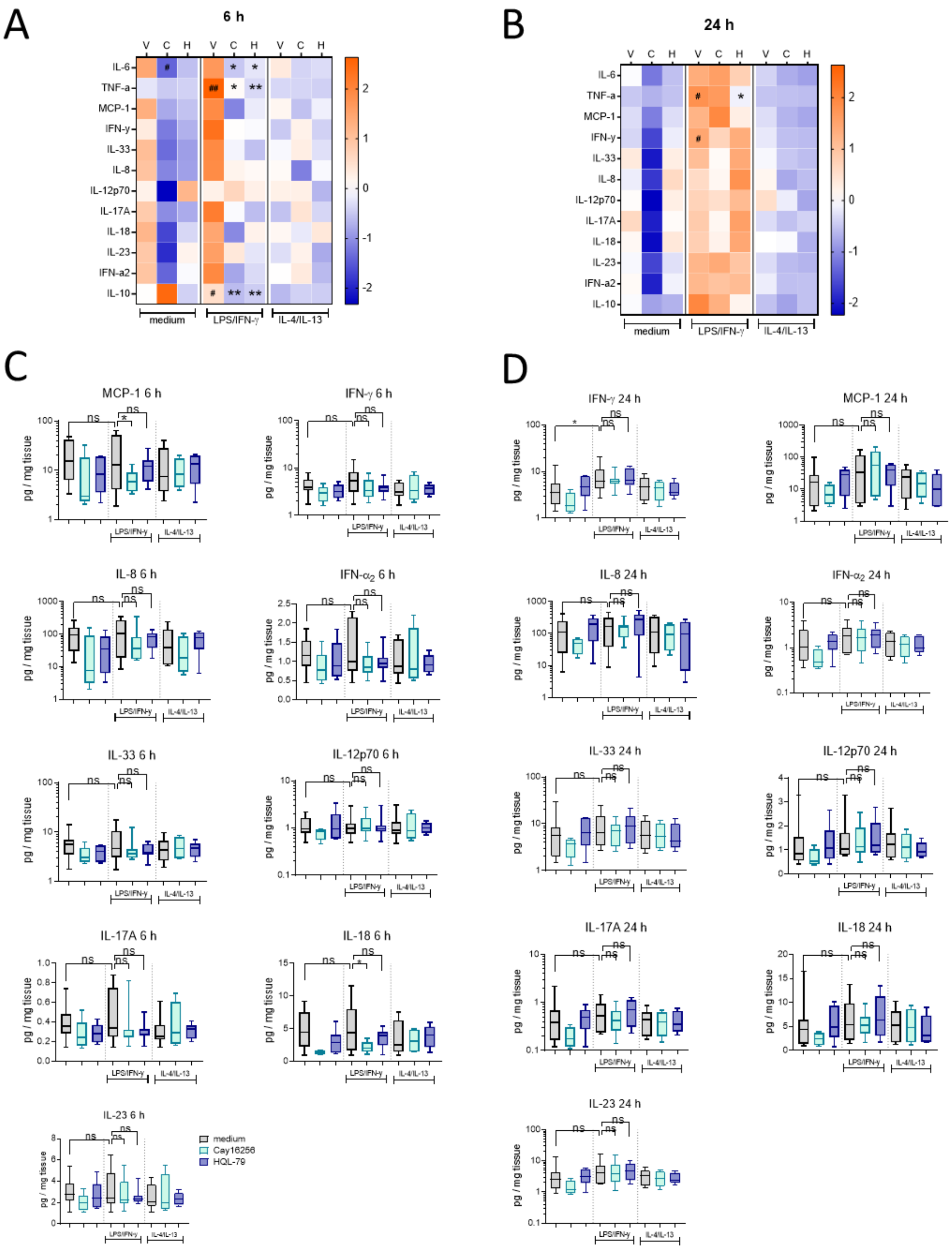

Figure S11. Collective LEGENDplex™ results for PCLS-conditioned medium. LPS/IFN-γ stimulation strongly increased the expression of type-1 pro-inflammatory cytokines, while (A) inhibition of hPGDS function counteracted this response for up to 6 h after activation. (B) 24 h after activation, hPGDS inhibition did no longer display a deterring effect. Individual graphs showing cytokine levels in conditioned medium normalized to tissue wet weight, collected after (C) 6h or (D) 24 h are shown. Data are presented as heatmap (z-scaled values) or box-and-whisker plot, n = 5-9, One-way ANOVA for repeated measurements (mixed model) with Sidak's *post hoc* test, A-B: V-vehicle, C-10 µM Cay16256, H-20 µM HQL-79, # depicts difference between medium/vehicle control and indicated condition, * depicts difference between LPS/IFN-γ/vehicle and indicated condition; C-D: * $p<0.05$.
